# Economic evaluation of sterilization reversal in infertility treatment: A systematic review

**DOI:** 10.1371/journal.pone.0350275

**Published:** 2026-06-01

**Authors:** Brandon Chongthanadon, Suvijak Untaaveesup, Chayanis Kositamongkol, Pochamana Phisalprapa, Krasean Panyakhamlerd, Vitaya Titapant

**Affiliations:** 1 Faculty of Medicine Siriraj Hospital, Mahidol University, Bangkok, Thailand; 2 Phaholpolpayuhasena Hospital, Kanchanaburi, Thailand; 3 Division of Ambulatory Medicine, Department of Medicine, Faculty of Medicine Siriraj Hospital, Mahidol University, Bangkok, Thailand; 4 Department of Obstetrics and Gynecology, Faculty of Medicine, Chulalongkorn University, Bangkok, Thailand; 5 Division of Maternal-Fetal Medicine, Department of Obstetrics and Gynecology, Faculty of Medicine, Siriraj Hospital, Mahidol University, Bangkok, Thailand; Medical Park Minatomirai, JAPAN

## Abstract

**Objectives:**

Although sterilization is intended to be permanent, some individuals later seek fertility. In such cases, options can be limited and financially burdensome. This review evaluated the cost-effectiveness of sterilization reversal surgery in previously sterilized individuals.

**Methods:**

We searched MEDLINE, Embase, and Scopus from inception in 1946 through December 2025, following the Preferred Reporting Items for Systematic Reviews and Meta-Analyses guidance. We included studies that analyzed cost-effectiveness or reported the costs of sterilization reversal in males (vasectomy reversal) or females (tubal anastomosis), with assisted reproductive technologies as comparators. Study quality was assessed using the Consolidated Health Economic Evaluation Reporting Standards 2022 checklist. Two authors independently screened each study to reduce bias. All costs per outcome were converted to 2024 United States dollars for analysis and comparisons.

**Results:**

Of 1628 identified articles, 24 studies met the eligibility criteria. Almost all examined populations in high-income countries, such as the United States, the Netherlands, and Singapore. Thirteen studies evaluated tubal anastomosis, and eleven evaluated vasectomy reversal. Most studies reported lower total costs for sterilization reversal than for assisted reproductive technologies, with comparable outcomes. Vasectomy reversal was preferred for male patients irrespective of the female partner’s age, whereas tubal anastomosis was preferred for female patients aged 40 years or younger. For older patients, assisted reproductive technologies were more cost-effective.

**Conclusions:**

Tubal anastomosis and vasectomy reversal may be economically advantageous compared with assisted reproductive technologies for infertility due to prior sterilization. However, societal factors, including a country’s socioeconomic context and policy feasibility, should be considered.

## Introduction

Surgical sterilization is a highly effective permanent contraceptive option, particularly attractive to individuals with a well-defined family plan. With no observed pregnancies within 6 months after vasectomy and a 2.9% pregnancy rate within 12 months after tubal sterilization, these sterilization techniques offer a reliable, long-term solution that often surpasses other methods in effectiveness and convenience [1,2].

Sterilization is classified into 2 categories: vasectomy in males and tubal sterilization in females. Vasectomy disrupts sperm transport from the proximal to the distal vas deferens, whereas tubal sterilization occludes the fallopian tube to prevent fertilization [3,4]. However, following the 2026 ESGO consensus statement, opportunistic salpingectomy has increasingly become the standard of care to significantly reduce the risk of tubo-ovarian carcinoma [5]. For individuals who initially opted for vasectomy or tubal sterilization, they may choose surgery or assisted reproductive technologies (ARTs) to regain fertility. On the other hand, if female individuals underwent salpingectomy, ARTs are the only options, as the procedure is anatomically impossible to be reversed. Surgical procedures to regain fertility include vasectomy reversal (VR) for males or tubal anastomosis (TA) for females. ARTs involve manipulation of oocytes or embryos and include in vitro fertilization (IVF), intracytoplasmic sperm injection (ICSI), and related procedures such as cryopreservation and donation of oocytes and embryos [6].

VR, introduced more than a century ago to restore fertility, is performed using 2 techniques [7]. Vasovasostomy anastomoses the severed ends of the vas deferens, whereas vasoepididymostomy (epididymovasostomy) joins the vas deferens to the epididymis. These procedures achieve pregnancy rates of 22%–68% (mean 49%) [8]. TA—also called tubal ligation reversal or tubal re-anastomosis—reconnects the ligated fallopian tube and has a pregnancy rate of 42%–69% [9]. In comparison, ART such as IVF has a pregnancy rate of approximately 46% [10]. However, TA is associated with a higher risk of ectopic pregnancy (6.7%) compared with both IVF (5.6%) and the general population (2%) [11].

Sterilization can lead to regret when life circumstances change and the desire to conceive returns. A retrospective analysis of National Survey of Family Growth data reported that approximately 10% of females regretted sterilization [12]. Without appropriate management, secondary infertility can impose a burden; therefore, addressing infertility in this population is important.

Cost-effectiveness is a key consideration when choosing between reversal surgery and ARTs. Multiple studies have compared sterilization reversal with ARTs; several reported favorable economic outcomes for reversal, whereas others favored ARTs. Reviews commonly concluded that VR was more cost-effective than ARTs [13,14], but findings for TA were variable [9]. However, comprehensive syntheses remain scarce, and many are narrative reviews without formal search methods, which can yield biased or misleading conclusions. The most recent systematic review reporting the cost-effectiveness of VR, conducted in 2002, emphasized ARTs rather than surgical reversal procedures [15].

To address this gap, this systematic review aimed to evaluate the cost-effectiveness of VR and TA compared with ARTs. Furthermore, by assessing studies without publication date restrictions, this review aims to evaluate the longitudinal shifts in cost-effectiveness driven by technological advancements in both surgeries and ARTs. We anticipate that the findings will inform evidence-based policy planning in infertility care.

## Materials and methods

### Literature search

We conducted a systematic literature search from inception date of each database through December 2025 in accordance with the Preferred Reporting Items for Systematic Reviews and Meta-Analyses protocol [16] (S1 Table). We searched MEDLINE (via PubMed, 1946–2025), Embase (1947–2025), and Scopus (1960–2025) following a protocol registered on PROSPERO (CRD420250588564) [17]. We also performed manual searches of reference lists of included studies and of relevant systematic reviews, narrative reviews, and meta-analyses. The detailed search strategy is provided in S2 Table.

### Inclusion criteria

We included economic evaluations (e.g., cost-effectiveness analyses, cost-utility analyses) of VR in males and TA in females, irrespective of language. The screening of non-English records was facilitated by a digital translation tool (i.e., Google Translate, Google LLC). We also considered clinical studies that reported economic outcomes as a prespecified outcome or secondary endpoint.

Eligible populations comprised individuals seeking to restore fertility after prior sterilization, regardless of sex or demographic characteristics. Populations could include patient cohorts from medical records or simulated cohorts in model-based analyses.

Interventions of interest were VR in males and TA in females, including vasovasostomy and vasoepididymostomy (epididymovasostomy) for VR and tubal ligation reversal or tubal re-anastomosis for TA. Comparators were ARTs, namely IVF, ICSI, and related procedures. We included studies comparing sterilization reversal with ARTs, regardless of terminology used.

### Exclusion criteria

We excluded studies of nonhuman subjects, as well as studies comparing variations of the intervention itself (e.g., 1-layer versus 2-layer VR or manual versus robotic TA). Additionally, studies focusing on same-sex couples were excluded. The primary objective of sterilization reversal is to restore fertility for unassisted reproduction. However, reproduction in same‑sex couples inherently requires ARTs or third-party gametes; therefore, evaluating sterilization reversal against ARTs is not clinically applicable in this cohort. We also excluded studies without full text or abstract, systematic or umbrella reviews, and studies using comparators that do not involve manipulation of oocytes or embryos, such as intrauterine insemination.

### Screening process

Two authors (B.C. and S.U.) independently screened titles and abstracts in Covidence, a web-based screening platform. Eligibility questions were addressed during screening; unresolved disagreements prompted full-text review and adjudication by a third author (C.K.). Only studies meeting all inclusion criteria proceeded to data extraction.

### Data extraction

One author (B.C.) extracted data, which a second author (S.U.) then cross-checked. Clinical variables included population demographics (country, sex, age); specified interventions; time horizon; and outcomes, including pregnancy, delivery, or live birth rates. Economic variables included intervention costs, discount rate, analytic perspective, cost per outcome, and the incremental cost-effectiveness ratio. When data were not specified, we recorded “not reported” in the extraction form. We recorded “not applicable” for fields that did not apply to a specific study design (e.g., time horizons in model analyses). We extracted each study’s conclusion on whether the intervention was more cost-effective than the comparator and categorized it as “yes,” “no,” or “inconclusive.”

### Data analysis

We categorized included studies into 2 groups based on biological sex: male and female. To compare costs across years, we adjusted each cost per outcome for inflation using the country’s healthcare consumer price index (CPI), standardizing to 2024 prices in the local currency. If a study did not report the reference year for currency conversion, we assumed the publication year. We then converted costs to 2024 United States dollars (US dollars, $) using the annual foreign exchange rate [18]. We reported results in a comparison table, categorized by participant sex and sorted by study year. We also stratified data by maternal age because this factor influences infertility [19]. For studies that did not report cost per outcome, i.e., those reporting only incremental costs, or only costs and outcomes separately, we included them in the analysis table as “not reported.” For studies reporting multiple interventional pathways, calculation was limited to isolated sterilization reversal arms and isolated ART arms to prevent confounding. Within each intervention category (VR or TA), the option with the lower inflation-adjusted cost per outcome was deemed more cost-effective.

### Study assessment

We assessed study quality using the Consolidated Health Economic Evaluation Reporting Standards (CHEERS) 2022 checklist, a structured framework for reporting economic evaluations [20]. Although developed as a reporting guideline, CHEERS is widely used to appraise health economic evaluations and was suitable for this review [21]. The checklist comprises 28 items spanning clinical and economic dimensions. We rated each item as fulfilled, not fulfilled, or not applicable. Studies meeting more CHEERS criteria were deemed to be of higher quality.

## Results

### Study selection

**Fig 1:**
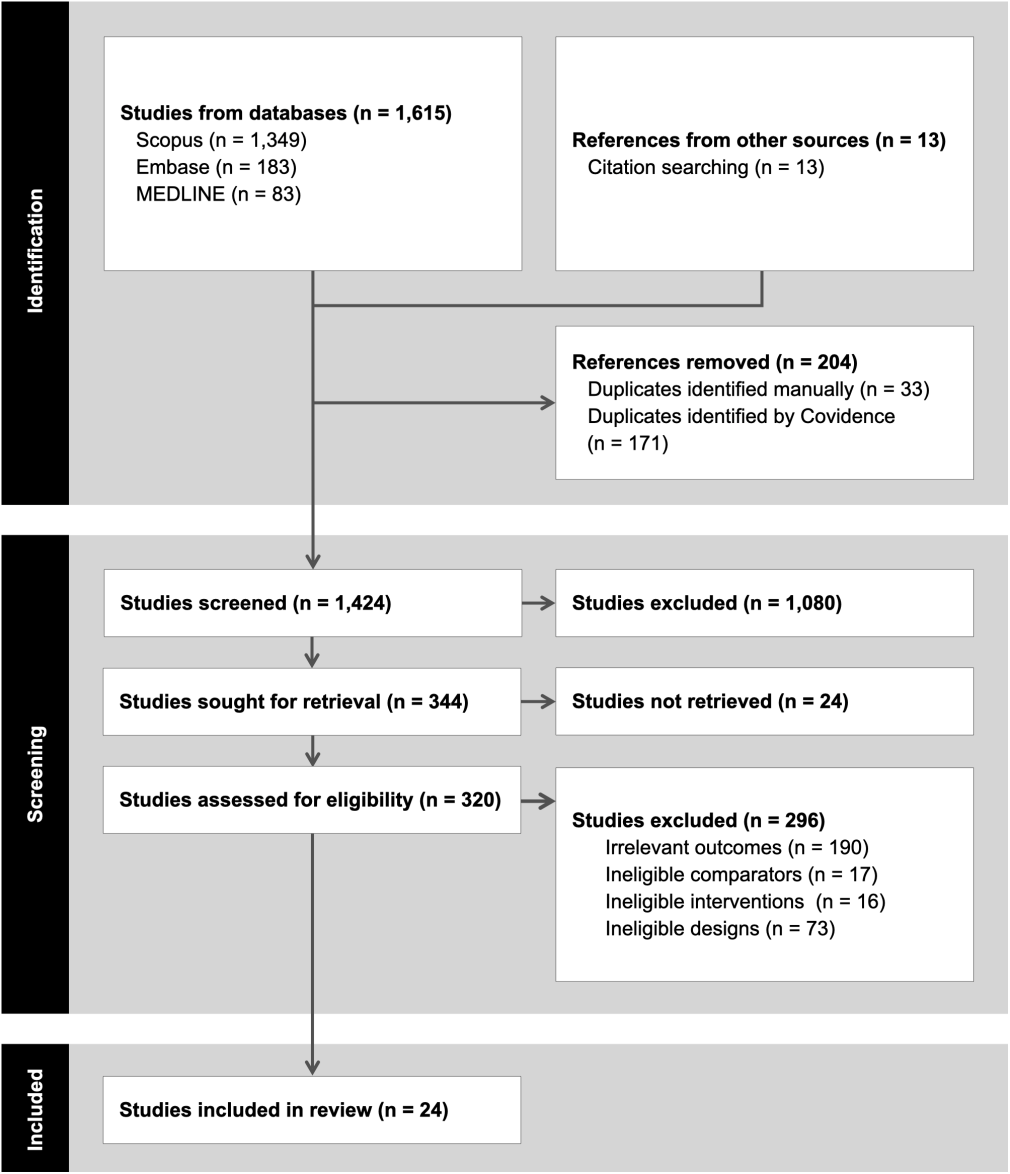
PRISMA diagram.

From the 3 databases, 1615 articles were identified. An additional 13 articles were found through citation searches, yielding 1628 in total. After removing 204 duplicates, 1424 titles and abstracts were screened. During screening, 1080 articles were excluded, leaving 344 articles for full-text retrieval. Of these, 24 were not retrieved due to lack of both full text and abstract, and 296 articles were excluded for the following reasons: 190 were excluded due to irrelevant outcomes; these were studies that did not report pregnancy, delivery, or live birth rates or any economic outcome; 17 were excluded due to ineligible comparators (i.e., studies that did not include ART as the comparator); 16 were excluded due to ineligible interventions; and 73 were excluded due to ineligible study designs, including non‑economic studies and those with ineligible populations. After full evaluation, 24 articles were included for data extraction and outcome interpretation (**Fig 1**).

### Study characteristics

Most studies (23 of 24) were conducted in high-income countries, with 16 from the United States. Two each were from the Netherlands [22,23] and Singapore [24,25], and 1 each from Australia [26], Belgium [27], Germany [28]. The remaining study [29] was conducted in China, the sole upper-middle-income country represented. Seven studies [22,23,30–34] were published between 1990–1999, two studies [28,35] were published between 2000–2002. Of these, only five studies [30,32–35] were included in the previous systematic review [15]; the remaining 15 studies were conducted after 2002.

### Study designs and evaluation types

**Fig 2:**
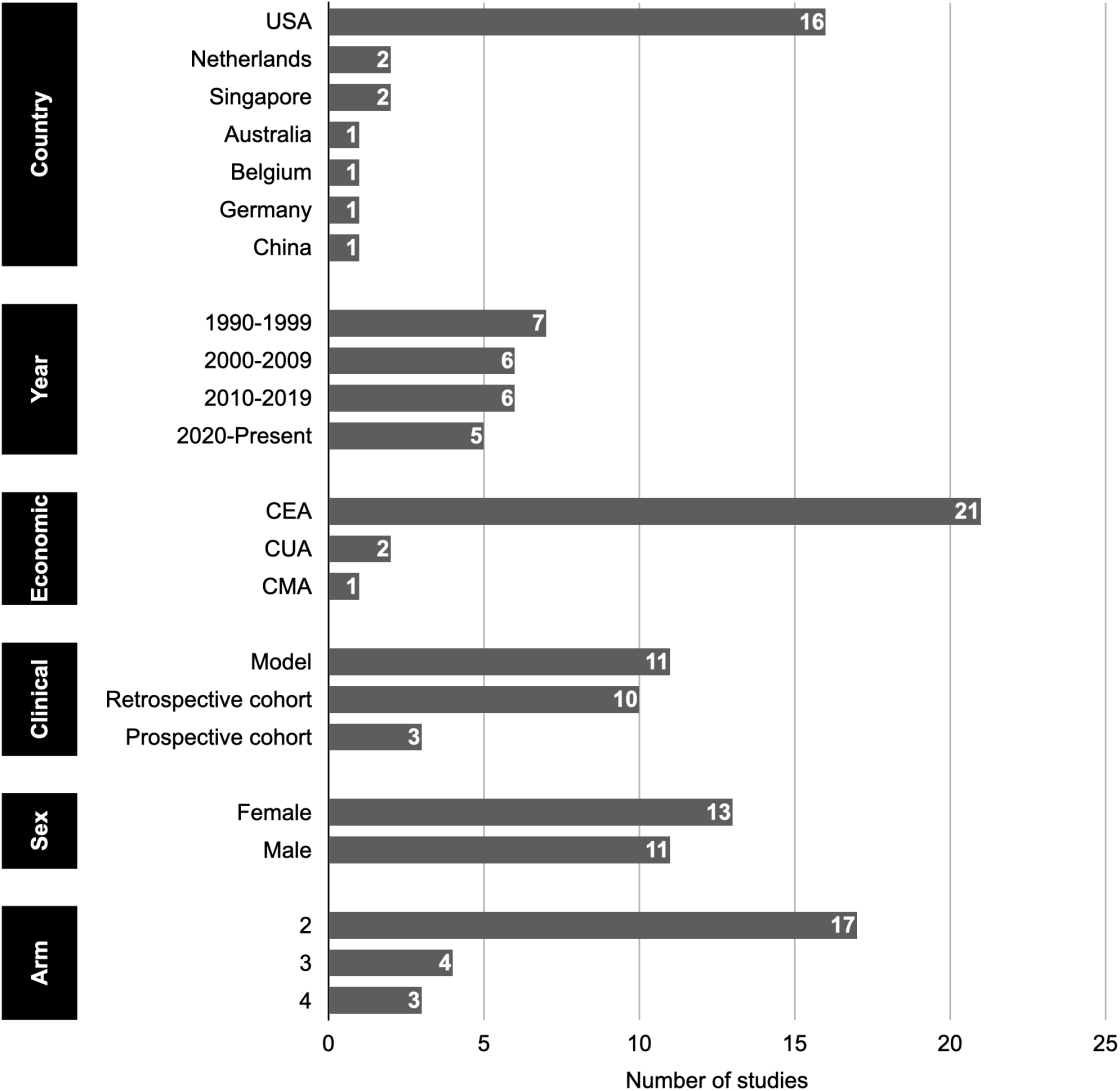
Characteristics of included studies.

Cost-effectiveness analyses predominated (*n* = 21), with 2 cost-utility analyses [36,37] and 1 cost-minimization analysis [29]. Model-based analyses were most common, accounting for 11 studies, followed by retrospective (*n* = 10) and prospective cohort (*n* = 3), respectively. Thirteen studies evaluated TA in females, and 11 evaluated VR in males. Most were 2-arm studies (*n* = 17), with fewer 3-arm (*n* = 4) and 4-arm (*n* = 3) designs (**Fig 2**).

### Time horizons and perspectives

Time horizons were reported primarily for cohort-based cost-effectiveness and cost-utility analyses. Nearly all cost-effectiveness analyses [22,24–28,30–33,35] and 1 cost-minimization analysis cohort study [29] reported time horizons of 2–11 years; 1 study did not report a horizon [23]. However, time horizons were marked as “not applicable” for static model-based analyses, as these specific studies evaluate costs and outcomes at a single point in time rather than tracking a cohort longitudinally. For studies’ perspective, only 6 of the 23 studies reported the analytic perspective [24,32,37–40]. Four used the patient perspective [24,32,37,38], 1 used the healthcare provider perspective [39], and 1 used the societal perspective [40]. Detailed characteristics are presented in **Table 1**.

**Table 1:** Characteristics of included studies.

Study (Authors, year)	Country	Design	Population	Maternal age (years)	Perspective	Time horizon	Intervention (number of patients)	Arm
**Tubal anastomosis**								
Alford et al, 2010* [38]	USA	CEA, model	Hypothetical patients	NR	Patient	NA	I: Bilateral TA	2
							C: IVF	
Boeckxstaens et al, 2007 [27]	Belgium	CEA, retrospective cohort	Patients from Dutch speaking Brussels Free University, Belgium	All age	NR	1990-2005 (6 years)	I: TA (*n* = 84)	2
							C: IVF (*n* = 79)	
Chua et al, 2020 [25]	Singapore	CEA, retrospective cohort	Previously ligated female patients	<40	NR	2011-2016 (6 years)	I: TA (*n* = 12)	2
							C: IVF (*n* = 31)	
Copperman et al, 1996 [30]	USA	CEA, prospective cohort	Female patients who underwent tubal surgery and has on concomitant male factor infertility.	<40	NR	1993-1994 (2 years)	I: TA (*n* = 31)	2
							C: IVF (*n* = 67)	
Haan et al, 1991 [22]	Netherlands	CEA, prospective cohort	Patients underwent IVF in 5 Dutch hospitals	All age	NR	1986-1988 (3 years)	I: TA	2
							C: Three cycles of IVF (*n* = 1462 for all IVF)	
Haan et al, 1992 [23]	Netherlands	CEA, prospective cohort	Patients underwent IVF in 5 Dutch hospitals	All age	NR	NR	I: TA	2
							C: IVF	
Hirshfeld-Cytron et al, 2013 [39]	USA	CEA, model	Hypothetical patients	All age	Healthcare provider	NA	I1: TA with more favorable prognosis (clip or ring tubal ligation)	3
							I2: TA with less favorable prognosis (postpartum tubal ligation, electrocautery, or unknown type)	
							I3: IVF	
Holst et al, 1991 [31]	USA	CEA, retrospective cohort	Female patients who underwent TA versus IVF	All age	NR	1980-1989 (10 years)	I1: TA in 1980–1982 (*n* = 206)	4
							I2: TA in 1986–1988 (*n* = 104)	
							C1: All IVF in 1986–1989 (*n* = 389)	
							C2: Completed IVF treatment in 1986–1989 (*n* = 128)	
Messinger et al, 2015 [40]	USA	CEA, model	Hypothetical patients	All age	Society	NA	I: TA	2
							C: IVF	
Petrucco et al, 2007 [26]	Australia	CEA, retrospective cohort	Female patients who underwent microsurgical TA	≥40	NR	1997-2005 (9 years)	I1: microsurgical TA in Adelaide, South Australia (*n* = 35)	3
							I2: microsurgical TA in Missouri, USA (*n* = 12)	
							C: IVF	
Tan et al, 2010 [24]	Singapore	CEA, retrospective cohort	Female patients who underwent TA without any known semen abnormalities	<40	Patient	1998-2008 (11 years)	I1: laparoscopic TA (*n* = 9)	3
							I2: open TA (n = 10)	
							C: IVF (*n* = 327)	
Winter et al, 2012* [41]	USA	CEA, model	Hypothetical patients	All age	NR	NA	I1: TA with more favorable prognosis (clip or ring tubal ligation)	3
							I2: TA with less favorable prognosis (postpartum tubal ligation, electrocautery, or unknown type)	
							I3: IVF	
Womack et at., 2020* [42]	USA	CEA, model	Hypothetical patients	NR	NR	NA	I: TA	2
							C: IVF	
**Vasectomy reversal**								
Cheng et al, 2021 [37]	USA	CUA, model	Hypothetical male patients with prior vasectomy	≥35	Patient	NA	I1: VR, then NC	4
							I2: SR, then IVF/ICSI	
							I3: back-up VR/SR, then IVF/ICSI, followed by NC	
							I4: back-up VR/SR, then NC, followed by IVF/ICSI	
Craig et al, 2017* [36]	USA	CUA, model	Hypothetical male patients with prior vasectomy	All age	NR	NA	I1: VR followed by NC	4
							I2: TESE followed by IVF	
							I3: back-up VR, then NC, followed by IVF	
							I4: back-up VR, then IVF, followed by NC	
Deck et al, 2000 [35]	USA	CEA, retrospective cohort	Male patients underwent microsurgical vasectomy reversal with ovulating female partner	>37	NR	1994-1998 (5 years)	I: Microsurgical VR (*n* = 29)	2
							C: TESE and IVF/ICSI	
Donovan et al, 1998 [32]	USA	CEA, retrospective cohort	Male patients with prior failed microscopic VR	All age	Patient	1986-1996 (11 years)	I: Repeat VR (*n* = 18)	2
							C: MESA and ICSI/IVF (*n* = 9)	
Heidenreich et al, 2000 [28]	Germany	CEA, retrospective cohort	Male patients who underwent microsurgical double-layer VVS for VR	All age	NR	1993-1998 (6 years)	I: VVS (*n* = 112)	2
							C: MESA/TESE and ICSI (*n* = 69)	
Kassab et al, 2024* [43]	USA	CEA, model	Hypothetical male patients with prior vasectomy	25-45	NR	NA	I: VR	2
							C: IVF	
Kolettis et al, 1997 [33]	USA	CEA, retrospective cohort	Male patients who underwent VES	All age	NR	1979-1995 (7 years)	I: VES (*n* = 55)	2
							C: MESA and ICSI	
Lee et al, 2008 [44]	USA	CEA, model	Hypothetical infertile male patients	All age	NR	NA	I: VR	2
							C: TESE/MESA and IVF	
Meng et al, 2005 [45]	USA	CEA, model	Hypothetical infertile male patients with varicocele or post-vasectomy obstruction	All age	NR	NA	I: VR	2
							C: SR and ICSI	
Pavlovich et al, 1997 [34]	USA	CEA, model	Hypothetical male patients with prior vasectomy	≤39	NR	NA	I: Microsurgical VR	2
							C: SR and ICSI	
Zhang et al, 2021 [29]	China	CMA, retrospective cohort	Male patients with obstructive azoospermia	<40	NR	2018-2019 (2 years)	I: microsurgical VES (*n* = 65)	2
							C: TESE/PESA with ICSI (*n* = 341)	

* Only abstracts were available.

**Abbreviations:** C, comparator; CEA, cost-effectiveness analysis; CMA, cost-minimization analysis; CUA, cost-utility analysis; I, intervention; ICSI, intracytoplasmic sperm injection; IVF, in vitro fertilization; MESA, microscopic epididymal sperm aspiration; n, number; NA, not applicable; NC, natural conception; NR, not reported; PESA, percutaneous epididymal sperm aspiration; SR, sperm retrieval; TA, tubal anastomosis; TESE, testicular sperm extraction; VES, vasoepididymostomy; VR, vasectomy reversal; VVS, vasovasostomy.

### Study populations

#### TA studies (female sterilization reversal).

Eight studies used patient records, with or without age restrictions [22–27,30,31]. Three reported results for females younger than 40 years [24,25,30]. Only 1 study reported results for females aged 40 years or older [26]. Three additional TA studies used hypothetical patients without age restrictions; all were model-based analyses [39–41]. Two other model-based analyses in the TA group did not specify population details [38,42].

#### VR studies (male sterilization reversal).

Almost half of the VR studies (*n* = 5) included real patients [28,29,32,33,35]. Among these, 1 study specified female partners older than 37 years [35], and 1 specified female partners younger than 40 years [29]. The remaining 6 studies modeled hypothetical male patients [34,36,37,43–45]. None reported male partner age; however, 1 specified female partners aged 35 years or older [37], 1 specified female partners aged 39 years or younger [34], and one stratified female partners aged from 25 to 45 years. Three studies specified the surgical technique: vasoepididymostomy [29,33] or vasovasostomy [28].

### Clinical and economic outcomes

#### TA outcomes.

Across TA studies [22–27,30,31,38–42], outcomes were reported as pregnancy, delivery, cumulative delivery, or live birth rates. As expected, pregnancy rates exceeded delivery and live birth rates, but values varied widely. Pregnancy rates for TA ranged from 5.0% [40] to 77.8% [24], compared with 10% [23,40] to 46.8% [24] for ARTs. Delivery rates in 1 study ranged from 36.6% to 72.2% for TA and from 51.4% to 52.4% for ARTs [27]. Live birth rates ranged from 23.7% [31] to 97.7% [39] for TA and from 12% [38] to 75.6% [39] for ARTs.

Almost all TA studies reported costs; however, 2 did not specify cost details [41,42]. Only 5 studies adjusted costs over time within their study periods [26,38–41].

#### VR outcomes.

VR studies reported pregnancy, delivery, and live birth rates; additionally, 2 reported quality-adjusted life years [36,37]. Eight VR studies reported total costs, either with a cost breakdown [28,32–34] or without detailed cost information [29,35,37,43]. Two model-based studies reported procedural costs only [44,45], and 1 did not specify cost details [36]. Only 1 study adjusted costs over time using a medical care CPI [44].

Details are summarized in **Table 2**.

**Table 2:** Interventions, outcomes, and costs.

Study (Authors, year)	Intervention	Source of outcomes	Outcomes	Source of costs	Conversion year	Discount rate	Costs
**Tubal anastomosis**							
Alford et al, 2010 [38]		Literature review	*Live birth rates by maternal age*	Literature review	2012	NR	*Total Cost*
	I: Bilateral TA		I: (<35) 47%, (41) 36%				I: $6551-$9593
	C: IVF		C: (<35) 41%, (41) 12%				C: $10 000-$21 000
Boeckxstaens et al, 2007 [27]		Institutional records	*Cumulative delivery rates (by maternal age)*	Literature review and institutional records	NR	NR	*Total cost*
	I: TA		I: average 59.5%, (<37) 72.2%, (≥37) 36.6%				I: medical cost of €2360, nonmedical cost of €1448
	C: IVF		C: average 52.0%, (<37) 52.4%, (≥37) 51.4%				C: medical cost of €2426, nonmedical cost of €575
Chua et al, 2020 [25]		Institutional records	*Pregnancy rates / live birth rates*	Institutional records	NR	NR	*Total cost*
	I: TA		I: 75.0% / 58.3%				I: SG$15 132
	C: IVF		C: 35.5% / 25.8%				C: Frozen embryo transfer SG$4000, fresh cycle SG$12 500, 31 fresh and 8 thaw cycles were performed
Copperman et al, 1996 [30]		Institutional records	*Pregnancy rates*	Institutional records	NR	NR	*Total cost*
	I: TA		I: 19.3%				I: $4696
	C: IVF		C: 25.0%				C: $5830
Haan et al, 1991 [22]		Institutional records	*Pregnancy rate*	Institutional records	NR	NR	*Total cost*
	I: TA		I: 30%				I: NLG 5500-NLG 7700
	C: Three cycles of IVF		C: 30%				C: NLG 2500 per cycle
Haan et al, 1992 [23]		Institutional records	*Pregnancy rate*	Institutional records	NR	NR	*Total cost*
	I: TA		I: 30%				I: NLG 5500-NLG 7700
	C: IVF		C: 10%				C: NLG 2500 per cycle
Hirshfeld-Cytron et al, 2013 [39]		Literature review	*Live birth rates (by maternal age)*	Literature review	2012	CPI	*Total cost (by maternal age)*
	I1: TA with more favorable prognosis		I1: (≤35) 97.7%, (35–40) 93.7%, (>40) 76.8%				I1: (≤35) $19 300, (35–40) $23 000, (>40) $26 200
	I2: TA with less favorable prognosis		I2: (≤35) 95.3%, (35–40) 91.9%, (>40) 74.3%				I2: (≤35) $20 700, (35 –40) $25 000, (>40) $27 200
	I3: IVF		I3: (≤35) 75.4%, (35–40) 75.6%, (>40) 48.5%				I3: (≤35) $26 200, (35 –40) $31 000, (>40) $28 900
Holst et al, 1991 [31]		Institutional records	*Live birth rates*	Institutional records	NR	NR	*Total cost*
	I1: TA in 1980–1982		I: 23.7%				I: $4000
	I2: TA in 1986–1988						
	C1: All IVF in 1986–1989		C: 72.3%				C: $1500 per cycle
	C2: Completed IVF treatment in 1986–1989						
Messinger et al, 2015 [40]		Literature review	*Ongoing pregnancy rates (by maternal age)*	Literature review	2014	CPI	*Total cost*
	I: TA		I: (<35) 63%, (35–40) 44%, (>40) 5%				I: $8685
	C: IVF		C: (<35) 40%, (35–40) 28%, (>40) 10%				C: $13 970
Petrucco et al, 2007 [26]		Institutional records	*Live birth rates*	Institutional records	2002, 2005	NR	*Total cost*
	I1: microsurgical TA in Adelaide		I: 40%				I1: AU$4850
	I2: microsurgical TA in Missouri						–
	C: IVF		C: NR				C: AU$6940
Tan et al, 2010 [24]		Institutional records	*Pregnancy rates / live birth rates*	Institutional records	NR	NR	*Group total cost*
	I1: laparoscopic TA		I1: 77.8% / 66.7%				I1: SG$85 200
	I2: open TA		I2: 70.0% / 60.0%				
	C: IVF		C: 46.8% / 34.6%				C: SG$2 649 402
Winter et al, 2012 [41]		Literature review	NR	Literature review	2012	NR	NR
	I1: TA with more favorable prognosis						
	I2: TA with less favorable prognosis						
	I3: IVF						
Womack et at., 2020 [42]		Literature review	NR	Institutional records	NR	NR	NR
	I: TA						
	C: IVF						
**Vasectomy reversal**							
Cheng et al, 2021 [37]		Literature review	*Live birth rates (by maternal age)* VR (35–37) 10.7–26.7%, (38–40) 7.8–19.5%, (>40) 4.6–11.5% IVF/ICSI (35–37) 28%, (38–40) 18.7%, (>40) 6.5% *Estimated QALY* Patient with a healthy child following fertility treatment: 0.93 Infertile patient with the desire for a child: 0.86	Institutional records	NR	NR	*Total cost*
	I1: VR, then NC						I1: $7150
	I2: SR, then IVF/ICSI						I2: $18 943
	I3: back-up VR/SR, then VF/ICSI, followed by NC						I3: $24 368
	I4: back-up VR/SR, then NC, followed by IVF/ICSI						I4: $24 368
Craig et al, 2017 [36]		Literature review	*QALY* Infertile couple: 0.56 Infertile couple who becomes pregnant: 0.63	Institutional records	NR	NR	NR
	I1: VR followed by NC						
	I2: TESE followed by IVF						
	I3: back-up VR, then NC, followed by IVF						
	I4: back-up VR, then IVF, followed by NC						
Deck et al, 2000 [35]		Institutional records	*Live birth rates*	Institutional records	NR	NR	*Total cost*
	I: Microsurgical VR		I: 17%				I: $4850
	C: TESE and IVF/ICSI		C: 8%				C: $8315 for TESE and 1 cycle of IVF/ICSI
Donovan et al, 1998 [32]		Institutional records	*Pregnancy rates / delivery rates*	Institutional records	NR	NR	*Total cost*
	I: Repeat VR		I: 44% / 44%				I: $9284
	C: MESA and ICSI/IVF		C: 67% / 56%				C: $17 092
Heidenreich et al, 2000 [28]		Institutional records	*Pregnancy rates / live birth rates*	Institutional records	NR	NR	*Total cost*
	I: VVS		I: 52% / 52%				I: (VVS) €2462
	C: MESA/TESE and ICSI		C: 24.5% / live birth rates (MESA/ICSI) 22.5% (TESE/ICSI) 19.5%				C: (MESA/TESE) €369, (ICSI) €2974
Kassab et al, 2024 [43]		Literature review	*Pregnancy rates by maternal age*	Literature review	NR	NR	*Total cost*
	I: VR		I: (25) 76.0%, (30) 67.1%, (35) 59.0%, (40) 39.3%, (42) 11.7%, (45) 11.7%				I: $12,558
	C: IVF		C: (25) 37.9%, (30) 37.9%, (35) 29.6%, (40) 20.1%, (42) 11.1%, (45) 3.5%				C: $27,647
Kolettis et al, 1997 [33]		Literature review and institutional records	*Pregnancy rates / delivery rates*	Institutional records	NR	NR	*Total cost*
	I: VES		I: 44% / 36%				I: $8500
	C: MESA and ICSI		C: 56% / 29%				C: $10 213
Lee et al, 2008 [44]		Literature review	*Pregnancy rates / delivery rate*	Literature review and institutional records	1999, 2005	Medical care CPI	*Procedural cost*
	I: VR		I: (VR patency) 81% / 42%				I: (VR) $2916
	C: TESE/MESA and IVF		C: (singleton) 70.4–72.0% (twin) 23.7–25.9% (triplet) 2.1–5.8% / 44%				C: (TESE) $577, (MESA) $1439, (IVF) $12 507
Meng et al, 2005 [45]		Literature review	*Patency rates* (Bilateral VVS) 87%, (VVS/VES) 70%, (bilateral VES) 65% *Pregnancy rates* (VR) 30%, (after 1 ICSI cycle) 30%, (after 4 IUI cycles) 32%	Institutional records	NR	NR	*Procedural cost* (Microsurgical VVS) $10 000, (microsurgical varicocelectomy) $4500, (IVF/ICSI cycle) $10 000, (IUI cycle) $500
	I: VR						
	C: SR and ICSI						
Pavlovich et al, 1997 [34]		Literature review	*Pregnancy rates / delivery rates*	Literature review and institutional records	NR	NR	*Total cost*
	I: Microsurgical VR		I: 52% / 47%				I: (VVS) $11 922, (VES) $17 151
	C: SR and ICSI		C: NR / 33%				C: (PESA/ICSI and TESE/ICSI) $20 347, (MESA/ICSI) $28 072
Zhang et al, 2021 [29]		Institutional records	*Pregnancy rates / delivery rates*	Institutional records	NR	NR	*Total cost*
	I: microsurgical VES		I: 44.6% / 38.5%				I: CN¥13 065
	C: TESE/PESA with ICSI		C: 27.6% / 25.8%				C: CN¥40 015

**Abbreviations:** $, US dollar; AU$, Australian dollar; C, comparator; CN¥, Chinese yuan; €, Euro; I, intervention; ICSI, intracytoplasmic sperm injection; IVF, in vitro fertilization; MESA, microscopic epididymal sperm aspiration; NC, natural conception; NLG, Dutch guilder; NR, not reported; PESA, percutaneous epididymal sperm aspiration; QALY, quality-adjusted life-year; SG$, Singapore dollar; SR, sperm retrieval; TA, tubal anastomosis; TESE, testicular sperm extraction; VES, vasoepididymostomy; VR, vasectomy reversal; VVS, vasovasostomy.

### Cost-effectiveness findings

#### Cost per outcome reporting.

In TA studies, cost-effectiveness was reported by clinical outcome; 2 studies reported incremental cost per live birth rather than cost per live birth [39,41]. In the VR group [28,29,32–37,43–45], nearly all studies reported cost per outcome; however, 1 cost-minimization analysis reported only intervention costs [29], and 1 cost-effective analysis reported only total costs [43].

#### TA findings.

In total, 9 of 13 studies concluded that TA was more cost-effective than ART. One study favored TA regardless of patient age or other characteristics [38]. However, 7 studies favored TA over ARTs for females below specific ages: below 37 years in 1 study [27], below 40 years in 3 studies [24,25,42], and below 41 years in 3 studies [39–41]. One study favored TA for females older than 40 years [26]. Two studies found ART to be as cost-effective as TA [22,23], and 2 favored ART over TA [30,31].

#### VR findings.

All VR studies concluded that VR was more cost-effective than ARTs for males seeking to restore fertility after prior vasectomy. Detailed cost-effectiveness data are presented in **Table 3**.

**Table 3:** Cost per outcome and study conclusions.

Study (Authors, year)	Intervention	Cost per outcome / ICER	Sensitivity analysis	Cost-effective?	Remark
**Tubal anastomosis**					
Alford et al, 2010 [38]		*Cost per delivery (by maternal age)*	Yes, not specified	Yes	–
	I: Bilateral TA	I: (<35) favored by $3980 - $37 281, (41) favored by $56 686-$156 803			
	C: IVF	C: base			
Boeckxstaens et al, 2007 [27]		*Cost per delivery (by maternal age)*	NR	Yes	For females patients <37 years old.
	I: TA	I: average €6015, (<37) €4953, (≥37) €9740			
	C: IVF	C: average €11 707, (<37) €12 140, (≥37) €11 214			
Chua et al, 2020 [25]		*Cost per live birth*	NR	Yes	For female patients <40 years old.
	I: TA	I: SG$27 109			
	C: IVF	C: SG$52 438			
Copperman et al, 1996 [30]		*Cost per pregnancy*	NR	No	For female patients <40 years old.
	I: TA	I: $24 334			
	C: IVF	C: $23 719			
Haan et al, 1991 [22]		*Cost per pregnancy*	NR	No conclusion	IVF appeared to be equivalently cost-effective as TA.
	I: TA	I: NLG 17 000-NLG 23 000			
	C: Three cycles of IVF	C: NLG 22 500			
Haan et al, 1992 [23]		*Cost per pregnancy*	NR	No conclusion	IVF appeared to be as cost-effective as TA.
	I: TA	I: equivalent to IVF			
	C: IVF	C: NLG 25 000			
Hirshfeld-Cytron et al, 2013 [39]		*ICER: Incremental cost per live birth (by maternal age)*	One-way analyses: varying ages and underlying diseases Two-way analyses: varying IVF cost and live birth rates	Yes	TA after a prior clip or ring tubal ligation for female patients ≤ 40 years old.
	I1: TA with more favorable prognosis	I1: (≤35) base, (35–40) base, (>40) base			
	I2: TA with less favorable prognosis	I2: (≤35) dominated, (35–40) dominated, (>40 years) dominated			
	I3: IVF	I3: (≤35) dominated by $30 900, (35–40) dominated by $44 000, (>40) dominated by $9540			
Holst et al, 1991 [31]		*Cost per live birth*	NR	No	–
	I1: TA in 1980–1982	I: $17 000			
	I2: TA in 1986–1988				
	C1: All IVF in 1986–1989	C: $12 000			
	C2: Completed IVF treatment in 1986–1989				
Messinger et al, 2015 [40]		*Cost per ongoing pregnancy (by maternal age)*	One-way analyses: varying IVF and TA charges	Yes	For female patients <41 years old.
	I: TA	I: (<35) $16 315, (35–40) $23 914, (>40) $218 742			
	C: IVF	C: (<35) $32 814, (35–40) $45 839, (>40) $111 445			
Petrucco et al, 2007 [26]		*Cost per live birth (by maternal age)*	NR	Yes	For female patients ≥40 years old.
	I1: microsurgical TA in Adelaide	I1: AU$11 317			
	I2: microsurgical TA in Missouri				
	C: IVF	C: (40–42) AU$97 884 (>42) AU$182 794			
Tan et al, 2010 [24]		*Cost per live birth*	NR	Yes	For female patients <40 years old.
	I1: laparoscopic TA	I1: SG$14 200			
	I2: open TA				
	C: IVF	C: SG$23 446			
Winter et al, 2012 [41]		*ICER: Incremental cost per live birth*	Yes, not specified	Yes	For female patients ≤ 40 years old with prior ring or clip ligation.
	I1: TA with more favorable prognosis	I1: Dominant			
	I2: TA with less favorable prognosis	I2: Additional $11 000-$60 000 per live birth			
	I3: IVF	I3: (younger cohort) additional $30 000-$45 000 per live birth, (oldest females) additional $200 000			
Womack et at., 2020 [42]		NR	Yes, not specified	Yes	For female patients <40 years old.
	I: TA				
	C: IVF				
**Vasectomy reversal**					
Cheng et al, 2021 [37]		*Cost per QALY (by maternal age)*	Adjusting live birth rates by maternal age	Yes	For male patients with female partners ≥35 years old.
	I1: VR, then NC	I1: (35–37) $7150, (38–40) $7203, (>40) $7367			
	I2: SR, then IVF/ICSI	I2: (35–37) $40 821, (38–40) $46 247, (>40) $54 599			
	I3: back-up VR/SR, then VF/ICSI, followed by NC	I3: (35–37) $31 289, (38–40) $33 226, (>40) $35 700			
	I4: back-up VR/SR, then NC, followed by IVF/ICSI	I4: (35–37) $34 142, (38–40) $35 404, (>40) $37 061			
Craig et al, 2017 [36]		*Cost per QALY (by maternal age)*	NR	Yes	–
	I1: VR followed by NC	I1: (<35) $11 349, (35–37) $11 350, (38–40) $11 485, (>40) $11 559			
	I2: TESE followed by IVF	I2: (<35) $54 719, (35–37) $59 340, (38–40) $65 749, (>40) $38 081			
	I3: back-up VR, then NC, followed by IVF	I3: (<35) $35 742, (35–37) $43 442, (38–40) $63 628, (>40) $41 826			
	I4: back-up VR, then IVF, followed by NC	I4: (<35) $36 803, (35–37) $38 176, (38–40) $38 350, (>40) $38 354			
Deck et al, 2000 [35]		*Cost per live birth*	NR	Yes	For male patients with female partners >37 years old.
	I: Microsurgical VR	I: $28 530			
	C: TESE and IVF/ICSI	C: $103 940			
Donovan et al, 1998 [32]		*Cost per pregnancy, cost per delivery*	Varying pregnancy and delivery rates of MESA and ICSI/IVF	Yes	–
	I: Repeat VR	I: $12 410, $14 892			
	C: MESA and ICSI/IVF	C: $25 637, $35 570			
Heidenreich et al, 2000 [28]		*Cost per live birth*	NR	Yes	–
	I: VVS	I: €2793			
	C: MESA/TESE and ICSI	C: €14 547			
Kassab et al, 2024 [43]		NR	NR	Yes	–
	I: VR				
	C: IVF				
Kolettis et al, 1997 [33]		*Cost per delivery, cost per live birth*	NR	Yes	–
	I: VES	I: $23 611, $31 099			
	C: MESA and ICSI	C: $35 217, $51 024			
Lee et al, 2008 [44]		*Cost per live birth in 1999 US dollars and 2005 US dollars*	One-way analyses: varying male procedural cost from high-volume andrology center and top quintile household income Two-way analyses: varying cost per live delivery over a range of VR patency rates and IVF delivery rates.	Yes	–
	I: VR	I: [1999] $19 633 | [2005] $20 903			
	C: TESE/MESA and IVF	C: (TESE) [1999] $45 637 | [2005] $54 797 (MESA) [1999] $48 055 | [2005] $56 861			
Meng et al, 2005 [45]		*Cost per pregnancy at 80% patency rate and 30% pregnancy rate*	One-way analyses: varying reversal patency rates at 40% constant pregnancy rate Two-way analyses: varying reversal patency and pregnancy rates	Yes	–
	I: VR	I: $38 983			
	C: SR and ICSI	C: $39 506			
Pavlovich et al, 1997 [34]		*Cost per delivery*	One-way analyses: varying delivery rates	Yes	For male patients with female partner ≤39 years old.
	I: Microsurgical VR	I: $25 475			
	C: SR and ICSI	C: $72 521			
Zhang et al, 2021 [29]		NR	NR	Yes	For male patients with female partner ≤40 years old.
	I: microsurgical VES				
	C: TESE/PESA with ICSI				

**Abbreviations:** $, US dollar; AU$, Australian dollar; €, Euro; C, comparator; I, intervention; ICER, incremental cost-effectiveness ratio; ICSI, intracytoplasmic sperm injection; IVF, in vitro fertilization; MESA, microscopic epididymal sperm aspiration; NC, natural conception; NLG, Dutch guilder; NR, not reported; PESA, percutaneous epididymal sperm aspiration; QALY, quality-adjusted life-year; SG$, Singapore dollar; SR, sperm retrieval; TA, tubal anastomosis; TESE, testicular sperm extraction; VES, vasoepididymostomy; VR, vasectomy reversal; VVS, vasovasostomy.

### Inflation-adjusted analyses

#### Adjustment methods.

Using the reported cost per outcome, we inflation-adjusted the costs from 17 studies to 2024 local currency values. We applied the Australian healthcare CPI [46], Belgian healthcare expenditure CPI [47], German healthcare CPI [48], Singaporean healthcare CPI [49], and United States medical care CPI [50]. We then converted values to 2024 US dollars using annual foreign exchange rates [18]. Calculation details, categorized by cost per outcome for comparison and stratified by maternal age, appear in S3 Table. Owing to substantial heterogeneity, we summarized findings in a table, as shown in Table 4, and performed no meta-analyses or sensitivity analyses.

**Table 4:** Cost per outcome of sterilization reversal versus assisted reproductive technology in 2024 US dollars.

Author, study year	Cost per outcome	Maternal age (years)										Cost-effective?*	Maternal age remark
		<35	35	36	37	38	39	40	41	42	>42		
**Tubal anastomosis**													
Haan, 1991 [22]	Cost per pregnancy	NR										Inconclusive	–
Holst, 1991 [31]	Cost per live birth	$54 149 / **$38 223**										No	–
Haan, 1992 [23]	Cost per pregnancy	NR										Inconclusive	–
Copperman, 1996 [30]	Cost per pregnancy	$60 107 / **$58 588**					NR					No	<40
Petrucco, 2005 [26]	Cost per live birth	NR					**$38 020** / $392 024			**$38 020** / $732 087		Yes	≥40
Boeckxstaens, 2007 [27]	Cost per delivery	**$4944** / $12 119	**$9723** / $11 194									Yes	<37
Alford, 2010 [38]	Cost per delivery	NR										Yes	–
Tan et al, 2010 [24]	Cost per live birth	**$14 342** / $23 680					NR					Yes	<40
Winter, 2012 [41]	Incremental cost per live birth	NR										Yes	≤40
Hirshfeld-Cytron, 2013 [39]	Incremental cost per live birth	NR										Yes	≤40
Messinger, 2015 [40]	Cost per pregnancy	**$21 132** / $42 503	**$30 975** / $59 374				$283 332 / **$144 352**					Yes	<41
Chua, 2020 [25]	Cost per live birth	**$22 766** / $44 037					NR					Yes	<40
Womack, 2020 [42]	Not reported	NR										Yes	<40
**Vasectomy reversal**													
Kolettis, 1997 [33]	Cost per delivery	**$56 749** / $74 747										Yes	–
Kolettis, 1997 [33]	Cost per live birth	**$74 747** / $122 636										Yes	–
Pavlovich, 1997 [34]	Cost per delivery	**$61 229** / $174 304					NR					Yes	≤39
Donovan, 1998 [32]	Cost per pregnancy	**$28 899** / $34 679										Yes	–
Donovan, 1998 [32]	Cost per delivery	**$59 701** / $82 833										Yes	–
Lee, 1999 [44]	Cost per live birth	**$44 181** / (TESE) $102 699, (MESA) $108 140										Yes	–
Deck, 2000 [35]	Cost per live birth	NR				**$61 693** / $224 758						Yes	>37
Heidenreich, 2000 [28]	Cost per live birth	**$3968** / $20 669										Yes	–
Lee, 2005 [44]	Cost per live birth	**$36 464** / (TESE) $95 589, (MESA) $99 190										Yes	–
Meng, 2005 [45]	Cost per pregnancy	**$68 003** / $68 915										Yes	–
Craig, 2017 [36]	Cost per QALY	**$13 463** / $64 910	**$13 464** / $70 392			**$13 624** / $77 994	**$13 712** / $45 173					Yes	–
Cheng, 2021 [37]	Cost per QALY	NR	**$7675** / $43 819			**$7732** / $49 644	**$7908** / $58 609					Yes	≥35
Zhang, 2021 [29]	Not reported	NR										Yes	≤40
Kassab, 2024 [43]	Not reported	NR										Yes	–

White cells indicate that sterilization reversal is more cost-effective, gray cells indicate that assisted reproductive technology is more cost-effective, while studies with black cells are not applicable for reporting economic outcome measured in cost per clinical outcome.

* As compared with assisted reproductive technologies.

**Abbreviations:** $, US dollars; NR, not reported; QALY, quality-adjusted life-year.

#### TA findings.

Studies conducted before 2000 favored ARTs over TA [30,31] or concluded that ARTs were as cost-effective as TA [22,23]. However, all studies conducted after 2000 concluded that TA was more cost-effective than ARTs for females younger than 40 or 41 years, with thresholds varying by study. One study reported higher cost per pregnancy for TA than for ARTs among females older than 40 years ($283 332 versus $144 352) [40].

#### VR findings.

VR was more cost-effective than ARTs in all studies, regardless of female partner age. Although two studies included multiple arms evaluating a combination of VR and ARTs [36,37], only isolated VR and ART arms were used for calculation. Cost estimates varied widely; however, 1 longitudinal analysis reported a lower cost per live birth for VR in 2005 than in 1999 [44].

### Assessment of study quality

**Fig 3:**
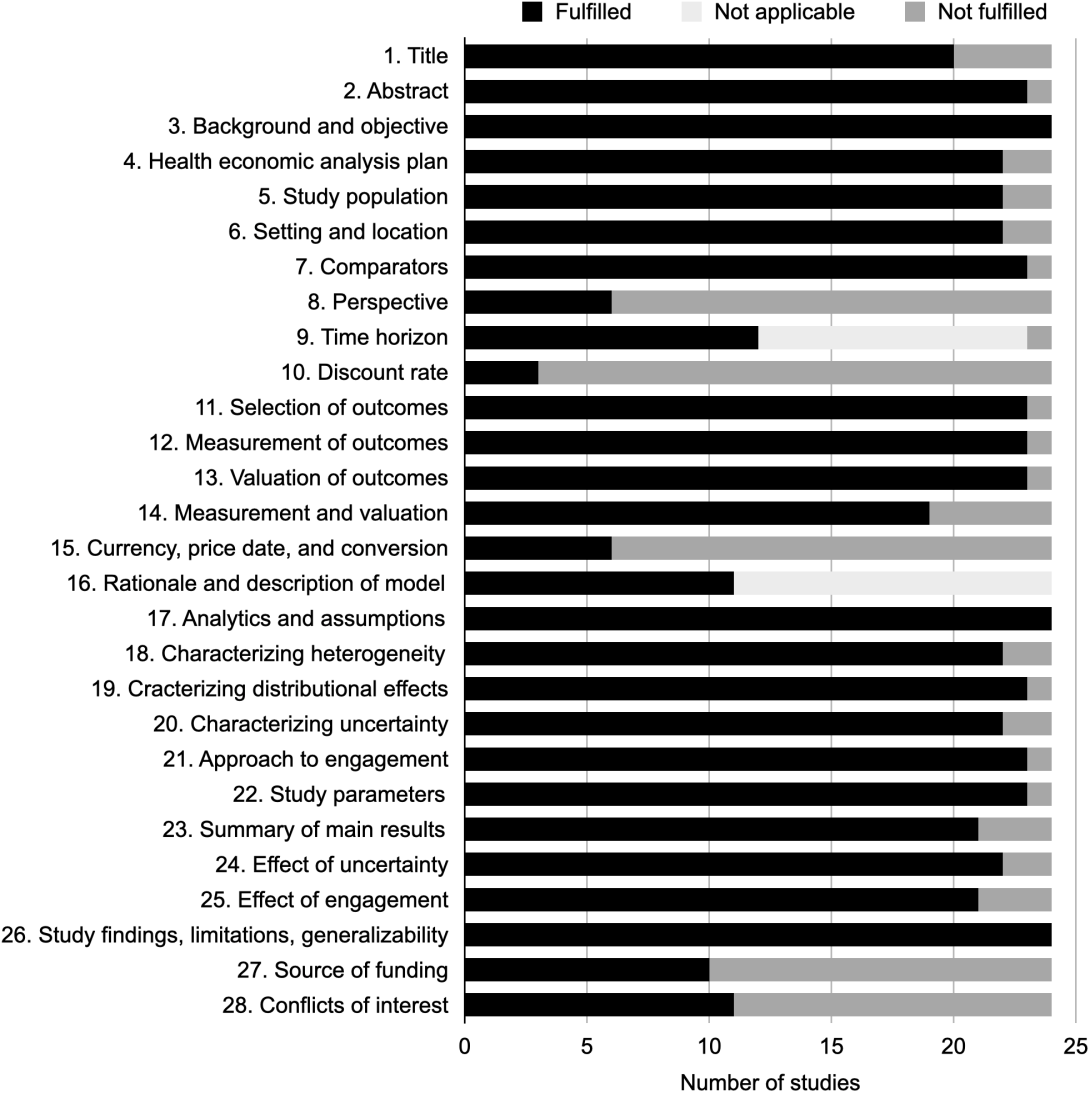
Visualization of Consolidated Health Economic Evaluation Reporting Standards 2022 checklist.

**Fig 3** summarizes CHEERS 2022 compliance by evaluation category. Almost all studies met more than 20 of 28 items; however, only 8 studies met at least 23 of 28 items (> 80%) [23,27,32,34,37,39,40,44]. One study met fewer than 10 items [42]. Six studies reported the year of currency conversion [26,38–41,44], and only 3 specified a discount rate [39,40,44]. Detailed study-level assessments appear in S4 Table.

## Discussion

Cost-effectiveness patterns differed by sex. In the TA group, studies conducted before 2000 reported ARTs to be either more cost-effective or as cost-effective as TA for achieving pregnancy. These findings align with the results of the previous systematic review published in 2002 [15]. In 1 study, the total cost of ART exceeded that of TA ($22 288.04 versus $20 039.74) [30]. However, the cost per pregnancy was slightly lower with ART ($23 718.80 versus $24 333.89) owing to a higher incidence of multiple gestations in the ART group. Despite higher total costs, multiple gestations reduced the cost per pregnancy. These findings aligned with the rapid expansion of ART availability after the first birth from IVF in 1978 [51].

After 2000, all studies favored TA over ARTs, particularly for females younger than 40 or 41 years, reflecting the sharp fertility decline after age 40. This growing favor toward TA in this younger cohort demonstrates a longitudinal shift in economic viability, likely driven by modern advancements in microsurgical techniques. Although ART success is substantially lower in this age group than in younger cohorts [52], 1 model-based analysis concluded that ART was more cost-effective than TA ($111 445 versus $218 742 per ongoing pregnancy) [40]. These findings suggest that ARTs are more economically viable for females of advanced maternal age; therefore, females in this age group should proceed directly to ARTs. Nevertheless, due to an increasing trend of opportunistic salpingectomy for the prophylaxis of tubo-ovarian carcinoma [5], a growing cohort of female patients lacks fallopian tubes required for re-anastomosis. For these individuals, ARTs are the only options to regain fertility, regardless of age.

VR studies demonstrated consensus despite differences in reported outcomes. Consistent with TA findings, 2 studies reported lower cost per outcome in male patients with younger female partners [36,37]. Nonetheless, costs varied widely by country, setting, and study year. After converting costs per outcome to 2024 US dollars (**Table 3** and S3 Table), VR generally demonstrated lower costs per outcome. One longitudinal study comparing VR and ART in 1999 and 2005 found improved cost-effectiveness over time, with VR remaining more cost-effective [44].

Therefore, rather than dictating clinical practice, the economic thresholds identified in this study provide an evidence base for shared decision-making. If anatomical reversal is possible, the data support counseling females over 40 years old to proceed directly with ARTs to prevent delay in care. Conversely, for the younger female cohorts, tubal anastomosis yields a superior economic return by allowing multiple unassisted reproduction attempts. Vasectomy reversal is preferred over ARTs regardless of age in male patients, as a single successful surgery provides a more cost-effective reproduction than a recurrent financial burden in ART cycles.

Despite the limited number of studies, substantial heterogeneity existed in reported results. Metrics varied, including cost per pregnancy, cost per delivery, and cost per live birth, with inconsistent application across studies. Even within the same intervention, there was no consensus on which outcome should serve as the denominator. Outcome selection may have favored measures that made the evaluated intervention appear cost-effective [53]. Consequently, an intervention with higher total cost might appear more cost-effective when evaluated using a different clinical outcome. Although cost per live birth is the definitive metric for clinical success, many studies reported only intermediate outcomes, such as cost per pregnancy. This likely reflects the logistical constraints of surgical cohort studies, where tracking initial postoperative conception is more feasible than maintaining the long-term follow-up required to confirm a live birth. In addition, several studies failed to report key components of economic evaluation, such as the analytic perspective, time horizon, and discount rates. Therefore, thorough evaluation should consider total costs, the chosen outcome measure, and other reported metrics.

Moreover, none of the included studies reported a stratification of male partners’ age. For studies evaluating tubal anastomosis, it was clinically sensible to factor in maternal age, as ovarian reserve predictably declines with advanced maternal age [54,55]. In contrast, although paternal age has been shown to negatively affect semen volume, motility, and morphology [56], a multivariate analysis revealed no significant difference in pregnancy rates after vasectomy reversal between males over and under 50 years old [57]. Therefore, while clinical pregnancy rates remain stable, future economic evaluations incorporating paternal age for vasectomy reversal could further address this gap.

Regarding cost adjustment, only 6 studies adjusted costs for inflation [26,38–41,44]. Of these, only 3 reported a discount rate [39,40,44]. Absent proper discounting, estimates drawn from different time periods may misstate the interventions’ economic value. Our systematic review addressed this limitation by standardizing all costs to 2024 US dollars, enabling direct comparisons and revealing trends across 4 decades.

Another limitation observed among primary economic evaluations is the geographic concentration of evidence. Nearly all studies were conducted in high-income countries, except for 1 study from China [29], which is the sole representative of upper-middle–income countries [58]. This distribution likely reflects disparities in access to reproductive technologies across socioeconomic settings, limiting generalizability to lower-income settings. Therefore, direct extrapolation of these findings to low- and middle-income countries should be done with considerable caution, as procedures may be structured differently. For instance, the relative costs of specialized surgical labor versus laboratory-intensive ART cycles are likely to follow different economic paradigms in resource-constraint settings. Additionally, an individuals’ true health financial burden is heavily influenced by income, local health policies, and insurance coverage. These are variables that cannot be retrospectively adjusted for in this analysis.

Furthermore, most included studies did not incorporate quality-adjusted life years into their analyses. Authors may have chosen outcome-specific metrics to allow readers to assess different dimensions of effectiveness without utility weighting [53]. However, the absence of quality-adjusted life years hindered aggregation of parental, fetal, and neonatal outcomes into a single metric and shifted focus to intermediate fetal outcomes rather than final neonatal health outcomes.

### Limitations

This systematic review focused exclusively on studies directly comparing sterilization reversal with ARTs, potentially excluding relevant cost data from other clinical or surgical contexts that could inform health economic decisions. Furthermore, given the scarcity of cost adjustments and the absence of paternal age stratification of included studies, future primary economic evaluations addressing these specific variables would provide a more comprehensive understanding of sterilization reversal costs. Moreover, although we performed an extensive citation search without language restrictions, some studies, especially those published in local languages, may have been overlooked because only English search terms were used.

Nevertheless, this review represents the most current and comprehensive synthesis of evidence on sterilization reversal; the last systematic review on this topic was published in 2002 [15]. All costs were standardized to 2024 US dollars, ensuring consistency and comparability across studies. The review also encompasses both male (VR) and female (TA) sterilization reversal. These strengths position this review as the most thorough and up-to-date resource available.

## Conclusions

Assuming anatomical feasibility, TA is cost-effective for restoring fertility, particularly for females younger than 40 years. For older females, ARTs are more cost-effective. Furthermore, for patients lacking fallopian tubes due to prior salpingectomy, ARTs remain the sole viable option, regardless of age. For males with prior vasectomy, VR is more cost-effective regardless of the female partner’s age. Therefore, sterilization reversal may confer an economic advantage over ARTs. However, owing to substantial methodological heterogeneity and the large proportion of evidence from high‑income countries, application of these findings requires careful consideration of local infrastructure, funding mechanisms, and health policy.

## Supporting information

S1 Table PRISMA 2020 checklist. (PDF)

S2 Table Search terms. (PDF)

S3 Table Cost per outcome of sterilization reversal in 2024 US dollars. * Health CPI of base year only. Only studies that reported costs per outcome were calculated. Abbreviations: ART, assisted reproductive technology; AUD, Australian dollar; CPI, consumer price index; EUR, Euro; LCU, local currency unit; NA, not applicable; QALY, quality-adjusted life-year; SGD, Singapore dollar; SR, sterilization reversal; USD, US dollar.
(PDF)

S4 Table Consolidated health economic evaluation reporting standards 2022 checklist. Abbreviations: N, “not fulfilled”; NA, “not applicable”; Y, “fulfilled.”.
(PDF)
