# Supplementary material for: Economic evaluation of sterilization reversal in infertility treatment: A systematic review
Source: PLoS One. 2026 Jun 1;21(6):e0350275. doi: 10.1371/journal.pone.0350275 (PMC13225410; doi:10.1371/journal.pone.0350275)
Supplement: S2 Table — (PDF) [file pone.0350275.s002.pdf]

S2 Table. Search terms

**MEDLINE**	
1	(infertility) OR (infertil*) OR (subfertility) OR (subfertil*)
2	(cost*) OR (economic*) OR (cost-effective*) OR (CEA) OR (cost-minimi*) OR (CMA) OR (cost-utili*) OR (CUA) OR (cost-benefit*) OR (CBA)
3	((vasectomy reversal) OR (vasovasostom*) OR (vaso-vasostom*) OR (vasoepididymostom*) OR (vaso-epididymostom*) OR (epididymovasostom*) OR (epididymo-vasostom*))
4	(tubal reversal) OR (tubal anastomos*) OR (tubal reanastomos*) OR (tubal re-anastomos*) OR (tubal ligation reversal) OR (tubal sterili* reversal)
5	#3 OR #4
6	#1 AND #2 AND #5
**Embase**	
1	('infertility'/exp OR infertility OR infertil* OR 'subfertility'/exp OR subfertility OR subfertil*)
2	(cost* OR economic* OR 'cost effective*' OR 'cea'/exp OR cea OR 'cost minimi*' OR cma OR 'cost utili*' OR cua OR 'cost benefit*' OR cba)
3	('vasectomy reversal'/exp OR 'vasectomy reversal' OR (('vasectomy'/exp OR vasectomy) AND reversal) OR vasovasostom* OR 'vaso vasostom*' OR vasoepididymostom* OR 'vaso epididymostom*' OR epididymovasostom* OR 'epididymo vasostom*' OR 'tubal reversal' OR (tubal AND reversal) OR (tubal AND anastomos*) OR (tubal AND reanastomos*) OR (tubal AND 're anastomos*') OR 'tubal ligation reversal' OR (tubal AND ('ligation'/exp OR ligation) AND reversal) OR 'tubal reversal' OR (tubal AND sterili* AND reversal))
4	#1 AND #2 AND #3
**Scopus**	
1	((infertility) OR (infertil*) OR (subfertility) OR (subfertil*))
2	((cost*) OR (economic*) OR (cost-effective*) OR (cea) OR (cost-minimi*) OR (cma) OR (cost-utili*) OR (cua) OR (cost-benefit*) OR (cba))
3	((vasectomy AND reversal) OR (vasovasostom*) OR (vaso-vasostom*) OR (vasoepididymostom*) OR (vaso-epididymostom*) OR (epididymovasostom*) OR (epididymo-vasostom*))
4	((tubal AND reversal) OR (tubal AND anastomos*) OR (tubal AND reanastomos*) OR (tubal AND re-anastomos*) OR (tubal AND ligation AND reversal) OR (tubal AND sterili* AND reversal))
5	#3 OR #4
6	#1 AND #2 AND #5
