# Supplementary material for: Economic evaluation of sterilization reversal in infertility treatment: A systematic review
Source: PLoS One. 2026 Jun 1;21(6):e0350275. doi: 10.1371/journal.pone.0350275 (PMC13225410; doi:10.1371/journal.pone.0350275)
Supplement: S3 Table — * Health CPI of base year only. Only studies that reported costs per outcome were calculated. Abbreviations: ART, assisted reproductive technology; AUD, Australian dollar; CPI, consumer price index; EUR, Euro; LCU, local currency unit; NA, not applicable; QALY, quality-adjusted life-year; SGD, Singapore dollar; SR, sterilization reversal; USD, US dollar. (PDF) [file pone.0350275.s003.pdf]

S3 Table. Costs per outcome of sterilization reversal in 2024 US dollars

Study	Maternal age	Country	LCU	Base year	CPI*	Cost in base year—LCU SR	Cost in base year—LCU ART	CPI* 2024	Cost in 2024—LCU SR	Cost in 2024—LCU ART	Exchange rate	Cost in 2024—USD SR	Cost in 2024—USD ART
**Tubal anastomosis**													
**Cost per pregnancy**													
**Copperman et al, 1996 (1)**	<40	USA	USD	1996	228.3	24 334	23 719	563.8	60 107	58 588	1.0000	60 107	58 588
**Messinger et al, 2015 (2)**	<35	USA	USD	2014	435.3	16 315	32 814	563.8	21 132	42 503	1.0000	21 132	42 503
	35-40	USA	USD	2014	435.3	23 914	45 839	563.8	30 975	59 374	1.0000	30 975	59 374
	>40	USA	USD	2014	435.3	218 742	111 445	563.8	283 332	144 352	1.0000	283 332	144 352
**Cost per delivery**													
**Boeckxstaens et al, 2007 (3)**	All	Belgium	EUR	2007	100.3	6015	11 707	108.4	6497	12 645	1.0820	6004	11 686
	<37	Belgium	EUR	2007	100.3	4953	12 140	108.4	5350	13 112	1.0820	4944	12 119
	≥37	Belgium	EUR	2007	100.3	9740	11 214	108.4	10 520	12 112	1.0820	9723	11 194
**Cost per live birth**													
**Chua et al, 2020 (4)**	<40	Singapore	SGD	2020	89.1	27 109	52 438	100.0	30 422	58 847	1.3363	22 766	44 037
**Holst et al, 1991 (5)**	NR	USA	USD	1991	177.0	17 000	12 000	563.8	54 149	38 223	1.0000	54 149	38 223
**Petrucco et al, 2007 (6)**	≥40	Australia	AUD	2005	74.8	11 317	NA	165.7	25 082	NA	0.6597	38 020	NA
	40-42	Australia	AUD	2002	62.7	NA	97 884	165.7	NA	258 618	0.6597	NA	392 024
	>42	Australia	AUD	2002	62.7	NA	182 794	165.7	NA	482 958	0.6597	NA	732 087
**Tan et al, 2010 (7)**	<40	Singapore	SGD	2010	74.1	14 200	23 446	100.0	19 165	31 644	1.3363	14 342	23 680
**Vasectomy reversal**													
**Cost per pregnancy**													
**Donovan et al, 1998 (8)**	NR	USA	USD	1998	242.1	12 410	14 892	563.8	28 899	34 679	1.0000	28 899	34 679
**Meng et al, 2005 (9)**	NR	USA	USD	2005	323.2	38 983	39 506	563.8	68 003	68 915	1.0000	68 003	68 915

Study	Maternal age	Country	LCU	Base year	CPI*	Cost in base year—LCU		CPI* 2024	Cost in 2024—LCU		Exchange rate	Cost in 2024—USD	
						SR	ART		SR	ART		SR	ART
**Cost per delivery**													
**Donovan et al, 1998 (8)**	NR	USA	USD	1998	242.1	25 637	35 570	563.8	59 701	82 833	1.0000	59 701	82 833
**Kolettis et al, 1997 (10)**	NR	USA	USD	1997	234.6	23 611	31 099	563.8	56 749	74 747	1.0000	56 749	74 747
**Pavlovich et al, 1997 (11)**	≤39	USA	USD	1997	234.6	25 475	72 521	563.8	61 229	174 304	1.0000	61 229	174 304
**Cost per live birth**													
**Deck et al, 2000 (12)**	>37	USA	USD	2000	260.8	28 530	103 940	563.8	61 693	224 758	1.0000	61 693	224 758
**Heidenreich et al, 2000 (13)**	NR	Germany	EUR	2000	70.1	2793	14 547	107.8	4294	22 364	1.0820	3968	20 669
**Kolettis et al, 1997 (10)**	NR	USA	USD	1997	234.6	31 099	51 024	563.8	74 747	122 636	1.0000	74 747	122 636
**Lee et al, 2008 (14)**	NR	USA	USD	1999	250.6	19 633	NA	563.8	44 181	NA	1.0000	44 181	NA
	NR	USA	USD	1999	250.6	NA	45 637	563.8	NA	102 699	1.0000	NA	102 699
	NR	USA	USD	1999	250.6	NA	48 055	563.8	NA	108 140	1.0000	NA	108 140
	NR	USA	USD	2005	323.2	20 903	NA	563.8	36 464	NA	1.0000	36 464	NA
	NR	USA	USD	2005	323.2	NA	54 797	563.8	NA	95 589	1.0000	NA	95 589
	NR	USA	USD	2005	323.2	NA	56 861	563.8	NA	99 190	1.0000	NA	99 190
**Cost per QALY**													
**Cheng et al, 2021 (15)**	35-37	USA	USD	2021	525.3	7150	40 821	563.8	7675	43 819	1.0000	7675	43 819
	38-40	USA	USD	2021	525.3	7203	46 247	563.8	7732	49 644	1.0000	7732	49 644
	>40	USA	USD	2021	525.3	7367	54 599	563.8	7908	58 609	1.0000	7908	58 609
**Craig et al, 2017 (16)**	<35	USA	USD	2017	475.3	11 349	54 719	563.8	13 463	64 910	1.0000	13 463	64 910
	35-37	USA	USD	2017	475.3	11 350	59 340	563.8	13 464	70 392	1.0000	13 464	70 392
	38-40	USA	USD	2017	475.3	11 485	65 749	563.8	13 624	77 994	1.0000	13 624	77 994
	>40	USA	USD	2017	475.3	11 559	38 081	563.8	13 712	45 173	1.0000	13 712	45 173

* Health CPI of base year only

Only studies that reported costs per outcome were calculated.

Abbreviations: ART, assisted reproductive technology; AUD, Australian dollar; CPI, consumer price index; EUR, Euro; LCU, local currency unit; NA, not applicable; QALY, quality-adjusted life-year; SGD, Singapore dollar; SR, sterilization reversal; USD, US dollar

References

1. Copperman AB, Mukherjee T, Shaer J, Patel D, Sandler B, Grunfeld L, et al. A Cost Analysis of In Vitro Fertilization Versus Tubal Surgery Within an Institution Under Two Payment Systems. Journal of Women's Health. 1996;5(4):335-41.

2. Messinger LB, Alford CE, Csokmay JM, Henne MB, Mumford SL, Segars JH, et al. Cost and efficacy comparison of in vitro fertilization and tubal anastomosis for women after tubal ligation. Fertil Steril. 2015;104(1):32-8.e4.

3. Boeckxstaens A, Devroey P, Collins J, Tournaye H. Getting pregnant after tubal sterilization: Surgical reversal or IVF? Hum Reprod. 2007;22(10):2660-4.

4. Chua KH, Chan JKY, Liu S, Tan TY, Phoon JWL, Viardot-Foucault VC, et al. Laparoscopic Tubal Re-anastomosis or In Vitro Fertilisation in Previously Ligated Patients: A Comparison of Fertility Outcomes and Survey of Patient Attitudes. Ann Acad Med Singap. 2020;49(4):180-5.

5. Holst N, Maltau JM, Forsdahl F, Hansen LJ. Handling of tubal infertility after introduction of in vitro fertilization: changes and consequences. Fertility and Sterility. 1991;55(1):140-3.

6. Petrucco OM, Silber SJ, Chamberlain SL, Warnes GM, Davies M. Live birth following day surgery reversal of female sterilisation in women older than 40 years: a realistic option in Australia? Med J Aust. 2007;187(5):271-3.

7. Tan HH, Loh SF. Microsurgical reversal of sterilisation - Is this still clinically relevant today? Ann Acad Med Singapore. 2010;39(1):22-6.

8. Donovan JF, Jr., DiBaise M, Sparks AE, Kessler J, Sandlow JI. Comparison of microscopic epididymal sperm aspiration and intracytoplasmic sperm injection/in-vitro fertilization with repeat microscopic reconstruction following vasectomy: is second attempt vas reversal worth the effort? Hum Reprod. 1998;13(2):387-93.

9. Meng MV, Greene KL, Turek PJ. Surgery or assisted reproduction? A decision analysis of treatment costs in male infertility. J Urol. 2005;174(5):1926-31; discussion 31.

10. Kolettis PN, Thomas AJ, Jr. Vasoepididymostomy for vasectomy reversal: a critical assessment in the era of intracytoplasmic sperm injection. J Urol. 1997;158(2):467-70.

11. Pavlovich CP, Schlegel PN. Fertility options after vasectomy: a cost-effectiveness analysis. Fertil Steril. 1997;67(1):133-41.

12. Deck AJ, Berger RE. Should vasectomy reversal be performed in men with older female partners? J Urol. 2000;163(1):105-6.

13. Heidenreich A, Altmann P, Engelmann UH. Microsurgical vasovasostomy versus microsurgical epididymal sperm aspiration/testicular extraction of sperm combined with intracytoplasmic sperm injection. A cost-benefit analysis. Eur Urol. 2000;37(5):609-14.

14. Lee R, Li PS, Goldstein M, Tanrikut C, Schattman G, Schlegel PN. A decision analysis of treatments for obstructive azoospermia. Human Reproduction. 2008;23(9):2043-9.

15. Cheng PJ, Kim J, Craig JR, Alukal J, Pastuszak AW, Walsh TJ, et al. "The Back-up Vasectomy Reversal." Simultaneous Sperm Retrieval and Vasectomy Reversal in the Couple With Advanced Maternal Age: A Cost-Effectiveness Analysis. Urology. 2021;153:175-80.

16. Craig J, Myers J, Brant W, Lenherr S, Walsh T, Alukal J, et al. “The back-up vasectomy reversal.” testicular sperm extraction at the time of vasectomy reversal in the couple with advanced maternal age: A cost-effectiveness analysis. Journal of Urology. 2017;197(4):e275.
