# Supplementary material for: Economic evaluation of sterilization reversal in infertility treatment: A systematic review
Source: PLoS One. 2026 Jun 1;21(6):e0350275. doi: 10.1371/journal.pone.0350275 (PMC13225410; doi:10.1371/journal.pone.0350275)
Supplement: S4 Table — Abbreviations: N, “not fulfilled”; NA, “not applicable”; Y, “fulfilled.”. (PDF) [file pone.0350275.s004.pdf]

S4 Table. Consolidated Health Economic Evaluation Reporting Standards 2022 checklist

	Tubal anastomosis													Vasectomy reversal										
	Alford et al, 2010 (1)	Boeckxstaens et al, 2007 (2)	Chua et al, 2020 (3)	Copperman et al, 1996 (4)	Haan et al, 1991 (5)	Haan et al, 1992 (6)	Hirshfeld-Cytron et al, 2013 (7)	Holst et al, 1991 (8)	Messinger et al, 2015 (9)	Petrucco et al, 2007 (10)	Tan et al, 2010 (11)	Winter et al, 2012 (12)	Womack et at., 2020 (13)	Cheng et al, 2021 (14)	Craig et al, 2017 (15)	Deck et al, 2000 (16)	Donovan et al, 1998 (17)	Heidenreich et al, 2000 (18)	Kassab et al, 2024 (19)	Kolettis et al, 1997 (20)	Lee et al, 2008 (21)	Meng et al, 2005 (22)	Pavlovich et al, 1997 (23)	Zhang et al, 2021 (24)
**Title**																								
1. Title	Y	Y	Y	Y	N	Y	Y	Y	Y	N	N	Y	Y	Y	Y	Y	Y	Y	Y	Y	Y	Y	Y	N
**Abstract**																								
2. Abstract	Y	Y	Y	Y	N	Y	Y	Y	Y	Y	Y	Y	Y	Y	Y	Y	Y	Y	Y	Y	Y	Y	Y	Y
**Introduction**																								
3. Background and objective	Y	Y	Y	Y	Y	Y	Y	Y	Y	Y	Y	Y	Y	Y	Y	Y	Y	Y	Y	Y	Y	Y	Y	Y
**Methods**																								
4. Health economic analysis plan	Y	Y	Y	Y	Y	Y	Y	Y	Y	N	Y	Y	Y	Y	Y	Y	Y	Y	Y	Y	Y	Y	Y	N
5. Study population	N	Y	Y	Y	Y	Y	Y	Y	Y	Y	Y	Y	N	Y	Y	Y	Y	Y	Y	Y	Y	Y	Y	Y
6. Setting and location	N	Y	Y	Y	Y	Y	Y	Y	Y	Y	Y	Y	N	Y	Y	Y	Y	Y	Y	Y	Y	Y	Y	Y
7. Comparators	Y	Y	Y	Y	Y	Y	Y	Y	Y	Y	Y	Y	N	Y	Y	Y	Y	Y	Y	Y	Y	Y	Y	Y
8. Perspective	Y	N	N	N	N	N	Y	N	Y	N	Y	N	N	Y	N	N	Y	N	N	N	N	N	N	N
9. Time horizon	NA	Y	Y	Y	Y	N	NA	Y	NA	Y	Y	NA	NA	NA	NA	Y	Y	Y	NA	Y	NA	NA	NA	Y
10. Discount rate	N	N	N	N	N	N	Y	N	Y	N	N	N	N	N	N	N	N	N	N	N	Y	N	N	N

	Tubal anastomosis													Vasectomy reversal										
	**Alford et al, 2010 (1)**	**Boeckxstaens et al, 2007 (2)**	**Chua et al, 2020 (3)**	**Copperman et al, 1996 (4)**	**Haan et al, 1991 (5)**	**Haan et al, 1992 (6)**	**Hirshfeld-Cytron et al, 2013 (7)**	**Holst et al, 1991 (8)**	**Messinger et al, 2015 (9)**	**Petrucco et al, 2007 (10)**	**Tan et al, 2010 (11)**	**Winter et al, 2012 (12)**	**Womack et at., 2020 (13)**	**Cheng et al, 2021 (14)**	**Craig et al, 2017 (15)**	**Deck et al, 2000 (16)**	**Donovan et al, 1998 (17)**	**Heidenreich et al, 2000 (18)**	**Kassab et al, 2024 (19)**	**Koletti s et al, 1997 (20)**	**Lee et al, 2008 (21)**	**Meng et al, 2005 (22)**	**Pavlovich et al, 1997 (23)**	**Zhang et al, 2021 (24)**
11. Selection of outcomes	Y	Y	Y	Y	Y	Y	Y	Y	Y	Y	Y	Y	N	Y	Y	Y	Y	Y	Y	Y	Y	Y	Y	Y
12. Measurement of outcomes	Y	Y	Y	Y	Y	Y	Y	Y	Y	Y	Y	Y	N	Y	Y	Y	Y	Y	Y	Y	Y	Y	Y	Y
13. Valuation of outcomes	Y	Y	Y	Y	Y	Y	Y	Y	Y	Y	Y	Y	N	Y	Y	Y	Y	Y	Y	Y	Y	Y	Y	Y
14. Measurement and valuation of resources and costs	Y	Y	Y	Y	Y	Y	Y	Y	Y	Y	Y	Y	N	Y	N	Y	Y	Y	N	Y	Y	N	Y	N
15. Currency, price date, and conversion	Y	N	N	N	N	N	Y	N	Y	Y	N	Y	N	N	N	N	N	N	N	N	Y	N	N	N
16. Rationale and description of model	Y	NA	NA	NA	NA	NA	Y	NA	Y	NA	NA	Y	Y	Y	Y	NA	NA	NA	Y	NA	Y	Y	Y	NA
17. Analytics and assumptions	Y	Y	Y	Y	Y	Y	Y	Y	Y	Y	Y	Y	Y	Y	Y	Y	Y	Y	Y	Y	Y	Y	Y	Y
18. Characterizing heterogeneity	Y	Y	Y	Y	Y	Y	Y	Y	Y	N	Y	Y	N	Y	Y	Y	Y	Y	Y	Y	Y	Y	Y	Y

	Tubal anastomosis													Vasectomy reversal										
	**Alford et al, 2010 (1)**	**Boeckxstaens et al, 2007 (2)**	**Chua et al, 2020 (3)**	**Copperman et al, 1996 (4)**	**Haan et al, 1991 (5)**	**Haan et al, 1992 (6)**	**Hirshfeld-Cytron et al, 2013 (7)**	**Holst et al, 1991 (8)**	**Messinger et al, 2015 (9)**	**Petrucco et al, 2007 (10)**	**Tan et al, 2010 (11)**	**Winter et al, 2012 (12)**	**Womack et at., 2020 (13)**	**Cheng et al, 2021 (14)**	**Craig et al, 2017 (15)**	**Deck et al, 2000 (16)**	**Donovan et al, 1998 (17)**	**Heidenreich et al, 2000 (18)**	**Kassab et al, 2024 (19)**	**Kolettis et al, 1997 (20)**	**Lee et al, 2008 (21)**	**Meng et al, 2005 (22)**	**Pavlovich et al, 1997 (23)**	**Zhang et al, 2021 (24)**
19. Characterizing distributional effects	Y	Y	Y	Y	Y	Y	Y	Y	Y	Y	Y	Y	N	Y	Y	Y	Y	Y	Y	Y	Y	Y	Y	Y
20. Characterizing uncertainty	Y	Y	Y	Y	Y	Y	Y	Y	Y	N	Y	Y	N	Y	Y	Y	Y	Y	Y	Y	Y	Y	Y	Y
21. Approach to engagement with patients and others affected by the study	N	Y	Y	Y	Y	Y	Y	Y	Y	Y	Y	Y	Y	Y	Y	Y	Y	Y	Y	Y	Y	Y	Y	Y
**Results**																								
22. Study parameters	Y	Y	Y	Y	Y	Y	Y	Y	Y	Y	Y	Y	N	Y	Y	Y	Y	Y	Y	Y	Y	Y	Y	Y
23. Summary of main results	N	Y	Y	Y	Y	Y	Y	Y	Y	Y	Y	N	N	Y	Y	Y	Y	Y	Y	Y	Y	Y	Y	Y
24. Effect of uncertainty	Y	Y	Y	Y	Y	Y	Y	Y	Y	N	Y	Y	N	Y	Y	Y	Y	Y	Y	Y	Y	Y	Y	Y
25. Effect of engagement with patients and others affected by the study	N	Y	Y	Y	Y	Y	Y	Y	Y	Y	Y	N	N	Y	Y	Y	Y	Y	Y	Y	Y	Y	Y	Y
**Discussion**																								

	Tubal anastomosis													Vasectomy reversal										
	Alford et al, 2010 (1)	Boeckxstaens et al, 2007 (2)	Chua et al, 2020 (3)	Copperman et al, 1996 (4)	Haan et al, 1991 (5)	Haan et al, 1992 (6)	Hirshfeld-Cytron et al, 2013 (7)	Holst et al, 1991 (8)	Messinger et al, 2015 (9)	Petrucco et al, 2007 (10)	Tan et al, 2010 (11)	Winter et al, 2012 (12)	Womack et at., 2020 (13)	Cheng et al, 2021 (14)	Craig et al, 2017 (15)	Deck et al, 2000 (16)	Donovan et al, 1998 (17)	Heidenreich et al, 2000 (18)	Kassab et al, 2024 (19)	Kolettis et al, 1997 (20)	Lee et al, 2008 (21)	Meng et al, 2005 (22)	Pavlovich et al, 1997 (23)	Zhang et al, 2021 (24)
26. Study findings, limitations, generalizability, and current knowledge	Y	Y	Y	Y	Y	Y	Y	Y	Y	Y	Y	Y	Y	Y	Y	Y	Y	Y	Y	Y	Y	Y	Y	Y
**Other relevant information**																								
27. Source of funding	Y	Y	N	N	Y	Y	Y	N	Y	Y	N	N	N	Y	N	N	Y	N	N	N	N	N	N	Y
28. Conflicts of interest	Y	N	N	N	Y	Y	Y	N	Y	Y	N	N	N	Y	N	N	Y	N	Y	N	N	N	Y	Y
**Summary**																								
Y	21	23	22	22	22	23	27	22	27	20	22	21	8	25	21	22	25	22	22	22	24	21	23	21
N	6	4	5	5	5	4	0	5	0	7	5	6	19	2	6	5	2	5	5	5	3	6	4	6
NA	1	1	1	1	1	1	1	1	1	1	1	1	1	1	1	1	1	1	1	1	1	1	1	1
% Fulfilling	75	82.1	78.6	78.6	78.6	82.1	96.4	78.6	96.4	71.4	78.6	75	28.6	89.3	75	78.6	89.3	78.6	78.6	78.6	85.7	75	82.1	75

Abbreviations: N, “not fulfilled”; NA, “not applicable”; Y, “fulfilled.”

References

1. Alford CE, Csokmay JM, Segars JH, Armstrong AY. Cost analysis of in vitro fertilization (IVF) versus bilateral tubal reanastomosis (BTA) to achieve a live birth. Fertility and Sterility. 2010;94(4):S34.
2. Boeckxstaens A, Devroey P, Collins J, Tournaye H. Getting pregnant after tubal sterilization: Surgical reversal or IVF? Hum Reprod. 2007;22(10):2660-4.
3. Chua KH, Chan JKY, Liu S, Tan TY, Phoon JWL, Viardot-Foucault VC, et al. Laparoscopic Tubal Re-anastomosis or In Vitro Fertilisation in Previously Ligated Patients: A Comparison of Fertility Outcomes and Survey of Patient Attitudes. Ann Acad Med Singap. 2020;49(4):180-5.
4. Copperman AB, Mukherjee T, Shaer J, Patel D, Sandler B, Grunfeld L, et al. A Cost Analysis of In Vitro Fertilization Versus Tubal Surgery Within an Institution Under Two Payment Systems. Journal of Women's Health. 1996;5(4):335-41.
5. Haan G. Effects and Costs of In-Vitro Fertilization: Again, Let's Be Honest. International Journal of Technology Assessment in Health Care. 1991;7(4):585-93.
6. Haan G, van Steen R. Costs in relation to effects of in-vitro fertilization. Human Reproduction. 1992;7(7):982-6.
7. Hirshfeld-Cytron J, Winter J. Laparoscopic tubal reanastomosis versus in vitro fertilization: Cost-based decision analysis. Am J Obstet Gynecol. 2013;209(1):56.e1-.e6.
8. Holst N, Maltau JM, Forsdahl F, Hansen LJ. Handling of tubal infertility after introduction of in vitro fertilization: changes and consequences. Fertility and Sterility. 1991;55(1):140-3.

9. Messinger LB, Alford CE, Csokmay JM, Henne MB, Mumford SL, Segars JH, et al. Cost and efficacy comparison of in vitro fertilization and tubal anastomosis for women after tubal ligation. Fertil Steril. 2015;104(1):32-8.e4.

10. Petrucco OM, Silber SJ, Chamberlain SL, Warnes GM, Davies M. Live birth following day surgery reversal of female sterilisation in women older than 40 years: a realistic option in Australia? Med J Aust. 2007;187(5):271-3.

11. Tan HH, Loh SF. Microsurgical reversal of sterilisation - Is this still clinically relevant today? Ann Acad Med Singapore. 2010;39(1):22-6.

12. Winter JA, Hirshfeld-Cytron JE. Laparoscopic tubal reanastomosis versus in vitro fertilization: Cost-based decision analysis incorporating multiple patientages, ligation technique and impact of multiple gestation. Fertility and Sterility. 2012;98(3):S36.

13. Womack AS, Tsang A, Buskmiller C, Mahnert ND, Keenan J, Avritscher E. Cost-Effectiveness Analysis of Tubal Surgery Versus in Vitro Fertilization As Treatment for Tubal Infertility. Journal of Minimally Invasive Gynecology. 2020;27(7):S143-S4.

14. Cheng PJ, Kim J, Craig JR, Alukal J, Pastuszak AW, Walsh TJ, et al. "The Back-up Vasectomy Reversal." Simultaneous Sperm Retrieval and Vasectomy Reversal in the Couple With Advanced Maternal Age: A Cost-Effectiveness Analysis. Urology. 2021;153:175-80.

15. Craig J, Myers J, Brant W, Lenherr S, Walsh T, Alukal J, et al. “The back-up vasectomy reversal.” testicular sperm extraction at the time of vasectomy reversal in the couple with advanced maternal age: A cost-effectiveness analysis. Journal of Urology. 2017;197(4):e275.

16. Deck AJ, Berger RE. Should vasectomy reversal be performed in men with older female partners? J Urol. 2000;163(1):105-6.

17. Donovan JF, Jr., DiBaise M, Sparks AE, Kessler J, Sandlow JI. Comparison of microscopic epididymal sperm aspiration and intracytoplasmic sperm injection/in-vitro fertilization with repeat microscopic reconstruction following vasectomy: is second attempt vas reversal worth the effort? Hum Reprod. 1998;13(2):387-93.

18. Heidenreich A, Altmann P, Engelmann UH. Microsurgical vasovasostomy versus microsurgical epididymal sperm aspiration/testicular extraction of sperm combined with intracytoplasmic sperm injection. A cost-benefit analysis. Eur Urol. 2000;37(5):609-14.

19. Kassab J, Mutascio C, Lipshultz L. (077) ECONOMIC ANALYSIS OF POST-VASECTOMY FERTILITY RESTORATION OPTIONS. The Journal of Sexual Medicine. 2024;21(Supplement_7).

20. Kolettis PN, Thomas AJ, Jr. Vasoepididymostomy for vasectomy reversal: a critical assessment in the era of intracytoplasmic sperm injection. J Urol. 1997;158(2):467-70.

21. Lee R, Li PS, Goldstein M, Tanrikut C, Schattman G, Schlegel PN. A decision analysis of treatments for obstructive azoospermia. Human Reproduction. 2008;23(9):2043-9.

22. Meng MV, Greene KL, Turek PJ. Surgery or assisted reproduction? A decision analysis of treatment costs in male infertility. J Urol. 2005;174(5):1926-31; discussion 31.

23. Pavlovich CP, Schlegel PN. Fertility options after vasectomy: a cost-effectiveness analysis. Fertil Steril. 1997;67(1):133-41.

24. Zhang Z, Zhang Y, Zhang N. Clinical outcome of microsurgical vasoepididymostomy versus epididymal or testicular sperm retrieval combined with intracytoplasmic sperm injection in obstructive azoospermia males. Andrologia. 2022;54(8).
