# Supplementary material for: Economic evaluation of sterilization reversal in infertility treatment: A systematic review
Source: PLoS One. 2026 Jun 1;21(6):e0350275. doi: 10.1371/journal.pone.0350275 (PMC13225410; doi:10.1371/journal.pone.0350275)
Supplement: Attachment — Submitted filename: EBC - Reviewer Report.docx [file pone.0350275.s005.docx]

**Reviewer Report: PONE-D-25-67814**

**Manuscript Title:** Economic evaluation of sterilization reversal in infertility treatment: A systematic review

**1. Introduction: Relevance and Methodological Rigor**

This systematic review addresses a significant gap in reproductive medicine, as the last comprehensive economic synthesis on this topic dates back to 2002. The manuscript demonstrates **commendable methodological rigor** by adhering to the **PRISMA 2020** guidelines and utilizing the **CHEERS 2022** checklist to assess the quality of economic evaluations.

A major technical strength is the standardization of historical costs across four decades into **2024 United States dollars (USD)** using local Healthcare Consumer Price Indices (CPI) . This allows for a more honest temporal comparison of the economic value of surgical reversal versus assisted reproductive technologies (ART).

**2. Evaluation of Sections**

**Title and Abstract**

- The title is clear and descriptive of the study's scope.
- The abstract follows the journal's structure, clearly outlining the objectives, search strategy (MEDLINE, Embase, Scopus), and the primary conclusion that reversal is generally more cost-effective for younger patients.

**Methodology**

- The use of a registered **PROSPERO** protocol (CRD420250588564) ensures transparency.
- **Statistical Note:** The authors correctly avoided a meta-analysis due to the extreme heterogeneity in study designs and reported metrics.

**Quality of References**

The bibliography includes high-impact journals such as *Human Reproduction Update* and *Fertility and Sterility*. However, there are notable concerns:

- **Geographic Bias:** 16 out of the 24 included studies originate from the United States. This limits the global applicability of the findings.
- **Economic Quality:** The authors acknowledge that only **8 of 24 studies** met more than 80% of the CHEERS criteria, indicating that the economic evidence base is of variable quality.
- **Technological Lag:** While the clinical references for ART are appropriate, the surgical references lack discussion on modern prophylactic paradigms.

**3. Critical Analysis and Major Concerns**

**A. Generalizability and LMIC Context**

The findings are heavily skewed toward high-income country data . The authors must add a paragraph cautioning against the direct extrapolation of these costs to **Low- and Middle-Income Countries (LMICs)**. In these regions, the valuation of health and the cost of specialized microsurgical labor versus laboratory-intensive ART cycles may follow entirely different economic structures.

**B. Outcome Inconsistency: Pregnancy vs. Live Birth**

There is a lack of standardization between "cost per pregnancy" and "cost per live birth" . For clinical counseling, the live birth rate is the only definitive metric for success. The authors should side-by-side these rates more explicitly to clarify if the economic advantage of surgery persists when only live births are considered.

**C. The Paradigm of Opportunistic Salpingectomy (Major Omission)**

The most critical clinical flaw is the failure to discuss **opportunistic salpingectomy**. Current international guidelines, including the **2026 ESGO consensus published in JAMA**, strongly advocate for total salpingectomy over traditional tubal ligation (salpingotripsy) for ovarian cancer prophylaxis.

- If total salpingectomy is performed as the standard for permanent contraception, **tubal anastomosis becomes anatomically impossible**.
- The authors must discuss how this shift toward non-reversible, cancer-preventive surgery impacts the future relevance of their economic model for female patients.

**4. Final Recommendation**

The manuscript is technically well-written and serves as a valuable historical and economic update. However, to be suitable for a high-impact journal like **PLOS ONE**, it must bridge the gap between economic theory and modern surgical-oncological reality.

**Recommendation: Major Revision**

The authors must:

1. Incorporate the **ESGO/JAMA 2026** paradigm on opportunistic salpingectomy.
2. Explicitly state the limitations of generalizing US-centric data to diverse global health systems.
3. Clarify the clinical weight of live birth rates versus intermediate pregnancy outcomes.
