# Supplementary material for: Economic evaluation of sterilization reversal in infertility treatment: A systematic review
Source: PLoS One. 2026 Jun 1;21(6):e0350275. doi: 10.1371/journal.pone.0350275 (PMC13225410; doi:10.1371/journal.pone.0350275)
Supplement: Attachment — Submitted filename: Rebuttal Letter 2026-04-22_CK.docx [file pone.0350275.s007.docx]

April 22, 2026

Dear Editors,

On behalf of all authors, I am pleased to submit our revised manuscript, “Economic evaluation of sterilization reversal in infertility treatment: A systematic review,” for consideration for publication in *PLOS ONE*.

We carefully addressed all comments and suggestions from the editor and reviewers. The manuscript has been thoroughly revised. In the accompanying point-by-point response, we provide detailed answers to each issue raised. We are grateful for the constructive feedback, which has improved the quality, transparency, and clarity of our work.

This manuscript is original, has not been published previously, and is not under consideration by any other journal. All authors contributed substantially to the study’s conception and design, data analysis and interpretation, and manuscript drafting and revision. Each author has reviewed and approved the final version. We declare no conflicts of interest.

This research was funded by the Royal Thai College of Obstetricians and Gynaecologists (grant number 011 RTCOG2309). The funders had no role in study design, data collection and analysis, decision to publish, or manuscript preparation. This study does not involve proprietary data, models, or methodologies. This manuscript is not under consideration by any other journal.

Thank you for considering our manuscript. We believe our findings align with the mission of *PLOS ONE* and will contribute meaningfully to the field of health economics and outcomes research.

Sincerely,

Chayanis Kositamongkol, PharmD, MSc

Corresponding Author
Division of Ambulatory Medicine, Department of Medicine
Faculty of Medicine Siriraj Hospital, Mahidol University
2 Wang Lang Road, Bangkok Noi
Bangkok 10700, Thailand
chayanis.kos@mahidol.ac.th

**Point-by-point responses**

**Additional Editor Comments:**

Thank you for your submission and for the thoughtful work presented in this manuscript. The paper has now been evaluated by five reviewers. One reviewer recommends acceptance, three recommend minor revisions, and one recommends major revisions.

After considering all reviewer comments and the overall quality and clarity of the manuscript, I believe that the concerns raised can be addressed with minor revisions. Therefore, I am inviting you to submit a revised version of the manuscript that carefully responds to all reviewer comments.

Please ensure that:

Each point raised by the reviewers is addressed in a point‑by‑point response document.

All clarifications, methodological details, and textual revisions are incorporated into the manuscript itself.

Any changes made in response to the reviewer who requested major revisions are clearly explained, especially where you believe only partial modification is appropriate.

Once the revised manuscript and response document are submitted, I will proceed with the next stage of the editorial assessment. At this time, no further external review is anticipated, provided that the revisions adequately address the reviewers’ concerns.

**Author response:** Thank you very much for considering our manuscript.

**Comments to the Author**

**A. Is the manuscript technically sound, and do the data support the conclusions?**

The manuscript must describe a technically sound piece of scientific research with data that supports the conclusions. Experiments must have been conducted rigorously, with appropriate controls, replication, and sample sizes. The conclusions must be drawn appropriately based on the data presented.

Reviewer #1: Partly

Reviewer #2: Yes

Reviewer #3: Yes

Reviewer #4: Yes

Reviewer #5: Yes

**Author response:** Thank you.

**B. Has the statistical analysis been performed appropriately and rigorously?**

Reviewer #1: N/A

Reviewer #2: Yes

Reviewer #3: Yes

Reviewer #4: Yes

Reviewer #5: N/A

**Author response:** Thank you.

**C. Have the authors made all data underlying the findings in their manuscript fully available?**

The [PLOS Data policy](http://track.editorialmanager.com/CL0/http:%252F%252Fwww.plosone.org%252Fstatic%252Fpolicies.action%2523sharing/1/010f019d3cbf1d3a-7c11f965-861f-416b-96a2-e1826a12c1b3-000000/SdoAHeRr2NhnKBOAgZxKJr9ahI4-HGOqVz6LVUkGR2E=256%22%20%5Ct%20%22_blank) requires authors to make all data underlying the findings described in their manuscript fully available without restriction, with rare exception (please refer to the Data Availability Statement in the manuscript PDF file). The data should be provided as part of the manuscript or its supporting information, or deposited to a public repository. For example, in addition to summary statistics, the data points behind means, medians and variance measures should be available. If there are restrictions on publicly sharing data—e.g. participant privacy or use of data from a third party—those must be specified.

Reviewer #1: Yes

Reviewer #2: Yes

Reviewer #3: Yes

Reviewer #4: Yes

Reviewer #5: Yes

**Author response:** Thank you.

**D. Is the manuscript presented in an intelligible fashion and written in standard English?**

PLOS ONE does not copyedit accepted manuscripts, so the language in submitted articles must be clear, correct, and unambiguous. Any typographical or grammatical errors should be corrected at revision, so please note any specific errors here.

Reviewer #1: Yes

Reviewer #2: Yes

Reviewer #3: Yes

Reviewer #4: Yes

Reviewer #5: Yes

**Author response:** Thank you.

**E. Review Comments to the Author**

Please use the space provided to explain your answers to the questions above. You may also include additional comments for the author, including concerns about dual publication, research ethics, or publication ethics. (Please upload your review as an attachment if it exceeds 20,000 characters)

**Reviewer #1**

**Q1.** “We excluded studies of same-sex couples and nonhuman subjects” - I find this sentence insensitive. Furthermore, I see no comprehensible reason why same-sex couples should be excluded.

**Author response:** We appreciate the opportunity to clarify our methodology. We have separated the mention of same-sex couples from nonhuman subjects in the revised manuscript.

Regarding the rational of exclusion, our core clinical question of our systematic review compares the cost effectiveness of sterilization reversal (to enable unassisted in vivo fertilization) versus assisted reproductive technologies (ARTs), with an outcome of interest being cost per pregnancy, delivery, and live birth. This comparison inherently requires a cohort of subjects for whom both present a viable anatomy for conception. However, unassisted in vivo fertilization is physiologically impossible for biologically same-sex couples. In such case, their family requires ARTs or third-party gametes. Therefore, evaluating sterilization reversal against ARTs is not clinically applicable in the cohort of same-sex couples. In addition, the highly variable costs of third-party reproduction could introduce substantial confounding and heterogeneity on the data, thereby potentially altering the economic outcomes.

**Revised text (Materials and Methods, Exclusion criteria):** “We excluded studies of nonhuman subjects, as well as studies comparing variations of the intervention itself (e.g., 1-layer versus 2-layer VR or manual versus robotic TA). Additionally, studies focusing on same-sex couples were excluded. The primary objective of sterilization reversal is to restore fertility for unassisted reproduction. However, reproduction in same‑sex couples inherently requires ARTs or third-party gametes; therefore, evaluating sterilization reversal against ARTs is not clinically applicable in this cohort.”

**Q2.** “We categorized included studies into 2 groups: male and female.” - If a distinction was made based on binary gender, I would also refer to it as such.

**Author response:** We appreciate the reviewer’s perspective regarding sociological gender constructs. This systematic review evaluated surgical interventions performed on specific reproductive anatomy (fallopian tube and vas deferens), our results are categorized by biological sex, not binary gender identity.

To ensure no other readers conflate biological sex with gender identity, we have ensured that the terms “male,” “female,” “male patients,” and “female patients” are used consistently instead of “men” and “women” throughout the manuscript to accurately reflect the anatomical and physiological parameters of the evaluated interventions. Additionally, we have clarified our methodology as shown below.

**Revised text (Materials and Methods, Data analysis):** “We categorized included studies into 2 groups based on biological sex: male and female.”

**Q3.** I find that the study selection section lacks explanations as to why certain studies were excluded. This is essential for reflecting on which studies may be missing in the discussion. This makes the research process less transparent.

**Author response:** We appreciate this feedback. Our predefined exclusion criteria were detailed in the exclusion criteria. Furthermore, while the numerical breakdown was detailed in the PRISMA diagram (Fig 1), we agree that the manuscript text benefits from this added context. We have updated the study selection section to explicitly state the exact counts and details of each exclusion category.

**Revised text (Results, Study selection):** “During screening, 1080 articles were excluded, leaving 344 articles for full-text retrieval. Of these, 24 were not retrieved due to lack of both full text and abstract, and 296 articles were excluded for the following reasons: 190 were excluded due to irrelevant outcomes; these were studies that did not report pregnancy, delivery, or live birth rates or any economic outcome; 17 were excluded due to ineligible comparators (i.e., studies that did not include ART as the comparator); 16 were excluded due to ineligible interventions; and 73 were excluded due to ineligible study designs, including non‑economic studies and those with ineligible populations.”

**Q4.** Adjustment methods; It is not only the cost of the procedure that is decisive. Due to the different situations of individuals in different healthcare systems, there are other factors that are decisive for the comparisons. For example, average income, the extent to which costs are covered by health insurance companies or similar institutions, and so on.

**Author response:** We appreciate the reviewer raising this point of concern under adjustment methods. As noted in the abstract, discussion, and conclusion of our manuscript, we fully agree that applying the results need a wider consideration of local policies, funding mechanism, and other socioeconomic factors. However, mathematically adjusting for these variables within our methodology is simply not feasible, as the reference studies did not report those specific costs in their calculation. Because this cannot be treated as a quantitative adjustment method, it is most appropriately handled as a structural limitation of the existing literature in this review. To ensure this limitation is completely unambiguous for readers, we have added a clarifying sentence to the discussion section as shown below.

**Revised text (Discussion):** “Additionally, an individuals’ true health financial burden is heavily influenced by income, local health policies, and insurance coverage. These are variables that cannot be retrospectively adjusted for in this analysis.”

**Q5.** The consequences of the results are not addressed sufficiently. What does it mean that IVF is made more economically accessible for older women? What does it mean that VR is cheaper for men with younger women?

**Author response:** We thank the reviewer for allowing us to clarify the clinical implications of our findings. First, as explicitly defined in our data analysis section, our methodology evaluated “cost-effectiveness” as represented by cost per outcome rather than “economic accessibility” or out-of-pocket affordability, which varied heavily by healthcare system. As we explained in data analysis section, we deemed intervention with lower cost per outcome as a more cost-effective option. In addition, as noted in our response to previous comments, true affordability varies substantially across healthcare systems and lies beyond the scope of a standard systematic review. Therefore, this required further consideration in various socioeconomics factors, as we already mentioned in several parts of our manuscript.

However, we agree that the clinical consequences of these cost-effective thresholds warrant further elaboration, regarding patient counseling. We have expanded our discussion section to include this clinical translation.

**Revised text (Discussion):** “Therefore, rather than dictating clinical practice, the economic thresholds identified in this study provide an evidence base for shared decision-making. If anatomical reversal is possible, the data support counseling females over 40 years old to proceed directly with ARTs to prevent delay in care. Conversely, for the younger female cohorts, tubal anastomosis yields a superior economic return by allowing multiple unassisted reproduction attempts. Vasectomy reversal is preferred over ARTs regardless of age in male patients, as a single successful surgery provides a more cost-effective reproduction than a recurrent financial burden in ART cycles.”

**Q6.** There is also a lack of limitation in that male age was not surveyed, and a lack of reflection on why this was probably not done.

**Author response:** We thank the reviewer for pointing this issue out. We have added a paragraph to the discussion section addressing this topic.

**Revised text (Discussion):** “Moreover, none of the included studies reported a stratification of male partners’ age. For studies evaluating tubal anastomosis, it was clinically sensible to factor in maternal age, as ovarian reserve predictably declines with advanced maternal age (55, 56). In contrast, although paternal age has been shown to negatively affect semen volume, motility, and morphology (57), a multivariate analysis revealed no significant difference in pregnancy rates after vasectomy reversal between males over and under 50 years old (58). Therefore, while clinical pregnancy rates remain stable, future economic evaluations incorporating paternal age for vasectomy reversal could further address this gap.”

**Q7.** There is a lack of classification of what knowledge could be generated and what is missing, due to the study designs of the included studies.

**Author response:** We thank the reviewer for this comment. We have added more clarification in limitation section regarding the evidence that remains missing.

**Revised text (Discussion):** “Furthermore, given the scarcity of cost adjustments and the absence of paternal age stratification of included studies, future primary economic evaluations addressing these specific variables would provide a more comprehensive understanding of sterilization reversal costs.”

**Reviewer #2**

**Q8.** Introduction: Relevance and Methodological Rigor

This systematic review addresses a significant gap in reproductive medicine, as the last comprehensive economic synthesis on this topic dates back to 2002. The manuscript demonstrates commendable methodological rigor by adhering to the PRISMA 2020 guidelines and utilizing the CHEERS 2022 checklist to assess the quality of economic evaluations.

A major technical strength is the standardization of historical costs across four decades into 2024 United States dollars (USD) using local Healthcare Consumer Price Indices (CPI). This allows for a more honest temporal comparison of the economic value of surgical reversal versus assisted reproductive technologies (ART).

**Author response:** Thank you.

**Q9.** Evaluation of Sections

Title and Abstract

- The title is clear and descriptive of the study's scope.
- The abstract follows the journal's structure, clearly outlining the objectives, search strategy (MEDLINE, Embase, Scopus), and the primary conclusion that reversal is generally more cost-effective for younger patients.

Methodology

- The use of a registered PROSPERO protocol (CRD420250588564) ensures transparency.
- Statistical Note: The authors correctly avoided a meta-analysis due to the extreme heterogeneity in study designs and reported metrics.
- Quality of References The bibliography includes high-impact journals such as Human Reproduction Update and Fertility and Sterility. However, there are notable concerns:
- Geographic Bias: 16 out of the 24 included studies originate from the United States. This limits the global applicability of the findings.
- Economic Quality: The authors acknowledge that only 8 of 24 studies met more than 80% of the CHEERS criteria, indicating that the economic evidence base is of variable quality.
- Technological Lag: While the clinical references for ART are appropriate, the surgical references lack discussion on modern prophylactic paradigms.

**Author response:** We thank the reviewer for the thorough evaluation and the positive feedback.

Firstly, we completely agree that the heavy reliance on US-based data limits global applicability. We have explicitly addressed this limitation in the discussion section.

**Revised text (Discussion):** “Another limitation observed among primary economic evaluations is the geographic concentration of evidence. Nearly all studies were conducted in high-income countries, except for 1 study from China (29), which is the sole representative of upper-middle–income countries (58). This distribution likely reflects disparities in access to reproductive technologies across socioeconomic settings, limiting generalizability to lower-income settings. Therefore, direct extrapolation of these findings to low- and middle-income countries should be done with considerable caution, as procedures may be structured differently. For instance, the relative costs of specialized surgical labor versus laboratory-intensive ART cycles are likely to follow different economic paradigms in resource-constraint settings.”

Secondly, to reflect the clinical reality of modern practice, we have incorporated the paradigm shift of opportunistic salpingectomy into our introduction and discussion sections.

**Revised text (Introduction):** “Sterilization is classified into 2 categories: vasectomy in males and tubal sterilization in females. Vasectomy disrupts sperm transport from the proximal to the distal vas deferens, whereas tubal sterilization occludes the fallopian tube to prevent fertilization (3, 4). However, following the 2026 ESGO consensus statement, opportunistic salpingectomy has increasingly become the standard of care to significantly reduce the risk of tubo-ovarian carcinoma (5). For individuals who initially opted for vasectomy or tubal sterilization, they may choose surgery or assisted reproductive technologies (ARTs) to regain fertility. On the other hand, if female individuals underwent salpingectomy, ARTs are the only options, as the procedure is anatomically impossible to be reversed.”

**Revised text (Discussion):** “Nevertheless, due to an increasing trend of opportunistic salpingectomy for the prophylaxis of tubo-ovarian carcinoma (5), a growing cohort of female patients lacks fallopian tubes required for re-anastomosis. For these individuals, ARTs are the only options to regain fertility, regardless of age.”

Lastly, we also expanded our Conclusion to emphasize this anatomical limitation.

**Revised text (Conclusios):** “Assuming anatomical feasibility, TA is cost-effective for restoring fertility, particularly for females younger than 40 years. For older females, ARTs are more cost-effective. Furthermore, for patients lacking fallopian tubes due to prior salpingectomy, ARTs remain the sole viable option, regardless of age.”

Critical Analysis and Major Concerns

**Q10.** Generalizability and LMIC Context

The findings are heavily skewed toward high-income country data. The authors must add a paragraph cautioning against the direct extrapolation of these costs to Low- and Middle-Income Countries (LMICs). In these regions, the valuation of health and the cost of specialized microsurgical labor versus laboratory-intensive ART cycles may follow entirely different economic structures.

**Author Response:** We thank the reviewer for highlighting this critical limitation. As detailed in our previous response regarding geographic bias, we entirely agree that direct extrapolation to low- and middle-income countries requires caution. In addition, we have also expanded our conclusion, as shown below.

**Revised text (Conclusions):** “However, owing to substantial methodological heterogeneity and the large proportion of evidence from high‑income countries, application of these findings requires careful consideration of local infrastructure, funding mechanisms, and health policy.”

**Q11.** Outcome Inconsistency: Pregnancy vs. Live Birth

There is a lack of standardization between "cost per pregnancy" and "cost per live birth". For clinical counseling, the live birth rate is the only definitive metric for success. The authors should side-by-side these rates more explicitly to clarify if the economic advantage of surgery persists when only live births are considered.

**Author response:** We thank the reviewer for highlighting this important clinical distinction. We agree that live birth is the definitive metric, and we have added text to the discussion section to address this disparity in reported outcomes.

**Revised text (Discussion):** “Although cost per live birth is the definitive metric for clinical success, many studies reported only intermediate outcomes, such as cost per pregnancy. This likely reflects the logistical constraints of surgical cohort studies, where tracking initial postoperative conception is more feasible than maintaining the long-term follow-up required to confirm a live birth.”

**Author response:** The reviewer also raises an excellent point regarding a side-by-side comparison. However, due to the high heterogeneity of the data (different denominators, years, and maternal age), we found that putting a direct side-by-side comparison in the main text would be difficult to comprehend. Therefore, to preserve the ability to observe longitudinal cost-effectiveness trends, we have retained the chronological sorting in **Table 4**. We have instead provided this specific categorized comparison within our supplementary data (**Table S2**) and added text directing readers to this breakdown.

**Revised text (Results,** **Inflation-adjusted analyses, Adjustment methods):** Calculation details, categorized by cost per outcome for comparison and stratified by maternal age, appear in **Table S2**.

**Q12.** The Paradigm of Opportunistic Salpingectomy (Major Omission)

The most critical clinical flaw is the failure to discuss opportunistic salpingectomy. Current international guidelines, including the 2026 ESGO consensus published in JAMA, strongly advocate for total salpingectomy over traditional tubal ligation (salpingotripsy) for ovarian cancer prophylaxis.

- If total salpingectomy is performed as the standard for permanent contraception, tubal anastomosis becomes anatomically impossible.
- The authors must discuss how this shift toward non-reversible, cancer-preventive surgery impacts the future relevance of their economic model for female patients.

**Author response:** We thank the reviewer for this suggestion. As detailed in our response to
Comment Q9, we discussed a limitation to tubal anastomosis in salpingectomy patients in our discussion section.

**Q13.** Final Recommendation

The manuscript is technically well-written and serves as a valuable historical and economic update. However, to be suitable for a high-impact journal like PLOS ONE, it must bridge the gap between economic theory and modern surgical-oncological reality.

Recommendation: Major Revision

The authors must:

- Incorporate the ESGO/JAMA 2026 paradigm on opportunistic salpingectomy.
- Explicitly state the limitations of generalizing US-centric data to diverse global health systems.
- Clarify the clinical weight of live birth rates versus intermediate pregnancy outcomes.

**Author response:** We thank the reviewer for these valuable suggestions, which have significantly strengthened the manuscript. As detailed in our responses to Comments Q9, Q10, and Q11 above, we have comprehensively updated the text to incorporate the 2026 ESGO consensus on opportunistic salpingectomy, explicitly state the limitations regarding the generalizability of US-centric data, and clarify the clinical importance of live birth rates compared to intermediate outcomes.

**Reviewer #3**

**Q14.** What was the date of inception of the data search? I.e. date of earliest data from MEDLINE, etc. Also, number of patients is small. Understand the limitations of the search but would benefit from more numbers to better help determine the cost-effectiveness. Also, would be good to see if there is more data in less well-developed areas to help validate the findings.

**Author response:** We appreciate the reviewer’s comment on inception date. Because the initial scoping search identified a scarcity of studies, we conducted our search from the inception of each database to ensure all available evidence was captured. We have also updated a literature search section to include all inception year of each database.

Regarding the small sample data size and underrepresentation of low-income and middle-income countries, we acknowledge this as a significant limitation. Despite exhaustive research, including both indexed database and from other sources, no studies meeting the inclusion criteria were identified from these countries. In addition, we have expanded our discussion to address the skew toward US-based healthcare costs, as suggested by **Reviewer #2**.

**Revised text (Abstract, Methods):** We searched MEDLINE, Embase, and Scopus from inception in 1946 through December 2025, following the Preferred Reporting Items for Systematic Reviews and Meta-Analyses guidance.

**Revised text (Materials and methods, Literature search):** We conducted a systematic literature search from inception date of each database through December 2025 in accordance with the Preferred Reporting Items for Systematic Reviews and Meta-Analyses protocol (17). We searched MEDLINE (via PubMed, 1946-2025), Embase (1947-2025), and Scopus (1960-2025) following a protocol registered on PROSPERO (CRD420250588564) (18).

**Reviewer #4**

The manuscript presents a systematic review on the economic evaluation of sterilization reversal in infertility treatment. The topic is relevant for reproductive health decision-making regarding fertility. The manuscript is generally well structured and has all the relevant supporting documents uploaded; however, a few reporting issues require clarification.

**Q15.** Clarification of intervention groups

Two of the included studies, Cheng et al 2021 (29) and Craig et al, 2017 (28), have the intervention group receiving sterilization (VR) followed by an artificial reproductive technique. This introduces potential confounding because the intervention group is exposed to two procedures. The authors should consider discussing its potential impact on the overall findings and conclusions

**Author response:** We thank the reviewer for this important observation. To ensure an unconfounded comparison, we extracted data only from the isolated VR and isolated ART arms of these studies. Numbers of these calculations are presented in **Table S2**. We also add more clarification on arm selection in data analysis section.

**Revised text (Materials and methods, Data analysis):** “For studies reporting multiple interventional pathways, calculation was limited to isolated sterilization reversal arms and isolated ART arms to prevent confounding.”

**Q16.** Unclear statement

The statement “However, time horizons marked as ‘not applicable’ for model analyses, which focus on evaluating at a single point in time” appears incomplete. The authors may wish to revise this sentence.

**Author response:** We thank the reviewer for identifying this incomplete phrasing. We have revised the sentence in the Results section (under “Time horizons and perspectives”) to improve clarity and grammatical completeness.

**Revised text (Results, Time horizons and perspectives):** “However, time horizons were marked as “not applicable” for static model-based analyses, as these specific studies evaluate costs and outcomes at a single point in time rather than tracking a cohort longitudinally.”

**Reviewer #5**

This is a review of the manuscript titled “Economic evaluation of sterilization reversal in infertility treatment: A systematic review”

My overall impression is that this is a well-conducted and well-written systematic review. This is an interesting contribution to the literature, however, it is suggested that a rationale of the inclusion of older studies and summary of their results from the previous review be made due to the known results from the prior systematic review. This SR could be reframed as an "update" and contribution to the literature.

This manuscript could be improved with the following recommendations:

**Q17.** Line 46: consider using either effectiveness for both vasectomy and tubal ligation, or pregnancy rate for both instead of comparing effectiveness with pregnancy rate to the reader.

**Author response:** We have revised the introduction to compare the pregnancy rates across all modalities.

**Revised text (Introduction):** With no observed pregnancies within 6 months after vasectomy and a 2.9% pregnancy rate within 12 months after tubal sterilization, these sterilization techniques offer a reliable, long-term solution that often surpasses other methods in effectiveness and convenience (1, 2).

**Q18.** Lines 57-63: good explanation of the reversals, consider adding complications such as ectopic pregnancy with TA, other pregnancy losses, etc.

**Author response:** We thank the reviewer for this suggestion. We have added risks of ectopic pregnancy in TA compared to IVF and general population in introduction section.

**Revised text (Introduction):** “However, TA is associated with a higher risk of ectopic pregnancy (6.7%) compared with both IVF (5.6%) and the general population (2%).”

**Q19.** Consider including the research question and hypothesis in the Introduction or Methods section to show why this study is unique and important, when the authors point out that a prior systematic review has been conducted in 2002.

**Author response:** We sincerely thank the reviewer for the assessment and thoughtful suggestion regarding framing of this study. We carefully discussed the possibility of reframing this manuscript as an update to the 2002 systematic review conducted by Garceau et al. However, as noted in our introduction, their primary objective was the economic evaluation of assisted reproductive techniques broadly. In that study, sterilization reversal was addressed only as a secondary comparator to ART. On the contrary, our study establishes sterilization reversal as the primary intervention of interest, meaning the foundational research questions and protocols differ significantly.

Therefore, we believe this work is more suitable to be presented as a stand-alone systematic review rather than an update to the 2002 review. To address the reviewer’s point, we also add a clearer rational for the inclusion of all studies without date restriction, allowing the evaluation of longitudinal shifts in cost-effectiveness trends over the past four decades in an introduction section.

**Revised text (Introduction):** “To address this gap, this systematic review aimed to evaluate the cost-effectiveness of VR and TA compared with ARTs. Furthermore, by assessing studies without publication date restrictions, this review aims to evaluate the longitudinal shifts in cost-effectiveness driven by technological advancements in both surgeries and ARTs. We anticipate that the findings will inform evidence-based policy planning in infertility care.”

**Q20.** Lines 53-56: ART and IVF and other procedure definitions are included, which is important, however, it seems these terms are used interchangeably throughout the manuscript based on the study. If ART includes IVF, consider using that language throughout to avoid confusion, as this is the term used in the abstract and conclusion.

**Author response:** Thank you for raising this concern, we have revised the manuscript to use the umbrella term “ART” throughout the manuscript, where appropriate, to avoid confusion as suggested.

**Q21.** Line 90: states “irrespective of language” Please clarify how the authors read/ comprehended publications not among those that they had literacy in.

**Author response:** We thank the reviewer for this methodological question. To minimize language bias, our initial search protocol did not apply any language filters. During the screening phase, we relied on the English abstracts, provided by international journals, supplemented by a digital translation tool (Google Translate) for titles or abstracts published exclusively in other languages.

However, following our rigorous screening process, all 24 studies that ultimately met our inclusion criteria for full-text data extraction were published in English. Therefore, full-text translation of non-English manuscripts was not required for this review. We have added a brief clarification to the methods section to ensure this is transparent to the reader.

**Revised text (Materials and methods, Inclusion criteria):** “We included economic evaluations (e.g., cost-effectiveness analyses, cost-utility analyses) of VR in males and TA in females, irrespective of language. The screening of non-English records was facilitated by a digital translation tool (i.e., Google Translate, Google LLC).”

**Q22.** Line 134: excellent use of the CHEERS checklist for this study

**Author response:** Thank you.

**Q23.** Line 154: consider mentioning that the only study included, conducted in a LMIC, was that of China

**Author response:** We have mentioned that one study was conducted in an upper-middle-income country. In addition, we would like to clarify that this point is addressed in the discussion section (Q10), where we explicitly state that nearly all studies were conducted in high-income countries, with the study from China (Zhang et al., 2021) being the sole representative from an upper-middle-income country.

**Revised text (Results, Study characteristics):** “Most studies (23 of 24) were conducted in high-income countries, with 16 from the United States. Two each were from the Netherlands (22, 23) and Singapore (24, 25), and 1 each from Australia (26), Belgium (27), Germany (28). The remaining study (29) was conducted in China, the sole upper-middle-income country represented.”

**Revised text (Discussion):** “Another limitation observed among primary economic evaluations is the geographic concentration of evidence. Nearly all studies were conducted in high-income countries, except for 1 study from China (29), which is the sole representative of upper-middle–income countries (58). This distribution likely reflects disparities in access to reproductive technologies across socioeconomic settings, limiting generalizability to lower-income settings.”

**Q24.** Line 234: the TA studies compared cost effectiveness to IVF and ART’s however the VR studies compared only cost effectiveness to ART’s, consider mentioning that comparison to IVF specifically was missing in the studies included or use ART as the overarching language throughout as mentioned previously.

**Author response:** We have updated the manuscript to use ART as the consistent umbrella term for the comparison group as suggested in Comment Q20.

**Q25.** Line 296: consider adding a sub-section starting here: “Limitations”

**Author response:** We have added a “Limitations” sub‑section at line 383, where we believe it is more appropriate.

**Q26.** Line 296: Cost per pregnancy: consider mentioning the possible outcomes: SAB, ectopic, preterm, live birth, etc., either here or in the Introduction, for the reader to be aware of the increased risk of ectopic pregnancy with TA.

**Author response:** Thank you. We have revised the Introduction, as shown in Comment Q18, for the reader to be aware of the increased risk of complications of TA.

**Q27.** Table 2 and Discussion section lines 326-27: Ten (10) out of the 24 studies included were from prior to 2002, which was when, as the authors point out, the last systematic review of the economic costs of sterilization reversal was conducted. Five (5) of these studies already reviewed, were also included in this review (Copperman, 1996; Deck, 2000; Donovan, 1998; Kolettis, 1997; and Palovich, 1997).

Consider giving rationale as to why were they included here. Consider also including years of the included studies (x before 2002 and Y since the prior systematic review) in the “Results” section.

Consider mentioning in the Limitations section, a comparison to prior findings from the 2002 article. Since the conclusions of the 2002 systematic review article according to the authors was that IVF/ ART was more cost effective (similar to your own older <2002 TA studies included here) how does including these articles in this review, contribute to the literature? Consider analyzing older studies separately.

**Author response:** We thank the reviewer for this insightful suggestion. As the reviewer noted, not all studies published before 2002 were included in the previous systematic review. By incorporating these older studies, we aim to provide a more comprehensive historical baseline for sterilization reversal. We have updated the results section to clarify the publication years of these older studies, and we have expanded the discussion to highlight how analyzing them chronologically demonstrates a longitudinal shift in cost-effectiveness over time.

**Revised text (Results, Study characteristics):** “Seven studies (22, 23, 30-34) were published between 1990-1999, two studies (28, 35) were published between 2000-2002. Of these, only five studies (30, 32-35) were included in the previous systematic review (15); the remaining 15 studies were conducted after 2002.”

**Revised text (Discussion):** “Cost-effectiveness patterns differed by sex. In the TA group, studies conducted before 2000 reported ARTs to be either more cost-effective or as cost-effective as TA for achieving pregnancy. These findings align with the results of the previous systematic review published in 2002 (15). …

After 2000, all studies favored TA over ARTs, particularly for females younger than 40 or 41 years, reflecting the sharp fertility decline after age 40. This growing favor toward TA in this younger cohort demonstrates a longitudinal shift in economic viability, likely driven by modern advancements in microsurgical techniques. Although ART’ success is substantially lower in this age group than in younger cohorts (52), 1 model-based analysis concluded that ART was more cost-effective than TA ($111 445 vs $218 742 per ongoing pregnancy) (40).”

**Q28.** Consider adding a statement at the conclusion of the manuscript stating whether AI was used in any of the analysis, writing, editing etc.

**Author response:** We appreciate the reviewer bringing up this important standard of transparency. We have added an artificial intelligence use declaration at the Acknowledgments section of our manuscript. Specifically, generative artificial intelligence (Gemini) was used strictly as an advanced language-editing tool to enhance readability and consolidate ideas. We confirm that artificial intelligence was not used in any part of the literature searching and screening, data extraction, economic analysis, quality assessment, or the formulation of scientific conclusions.

**Revised text (Acknowledgments):** “Artificial intelligence was not utilized for literature searching and screening, data collection, analysis, or critical appraisal of the included literature. During the manuscript preparation, the authors used Gemini (Google) to improve language readability and consolidate structural ideas. After using this tool, the authors and a native‑English‑speaking medical‑manuscript editor reviewed and edited the manuscript as needed. The authors take full responsibility for the final content of the publication.”

**F. PLOS authors have the option to publish the peer review history of their article (**[**what does this mean?**](https://track.editorialmanager.com/CL0/https:%252F%252Fjournals.plos.org%252Fplosone%252Fs%252Feditorial-and-peer-review-process%2523loc-peer-review-history/1/010f019d3cbf1d3a-7c11f965-861f-416b-96a2-e1826a12c1b3-000000/EfioCsXCEvRkXKaZAsPvI7vb7P3WHT7UiiNJ1JFskXg=256%22%20%5Ct%20%22_blank)**). If published, this will include your full peer review and any attached files.**

If you choose “no”, your identity will remain anonymous but your review may still be made public.

**Do you want your identity to be public for this peer review?** For information about this choice, including consent withdrawal, please see our [Privacy Policy](https://track.editorialmanager.com/CL0/https:%252F%252Fwww.plos.org%252Fprivacy-policy/1/010f019d3cbf1d3a-7c11f965-861f-416b-96a2-e1826a12c1b3-000000/bNsiSRWyvAu-7UxHqJ6pdg9fHBzYPI1-xaZoUYAlYic=256%22%20%5Ct%20%22_blank).

Reviewer #1: **Yes:** Lisa Glaum

Reviewer #2: No

Reviewer #3: No

Reviewer #4: No

Reviewer #5: No

[NOTE: If reviewer comments were submitted as an attachment file, they will be attached to this email and accessible via the submission site. Please log into your account, locate the manuscript record, and check for the action link "View Attachments". If this link does not appear, there are no attachment files.]

To ensure your figures meet our technical requirements, please review our figure guidelines: [https://journals.plos.org/plosone/s/figures](https://journals.plos.org/plosone/s/figures%22%20%5Ct%20%22_blank)

You may also use PLOS’s free figure tool, NAAS, to help you prepare publication quality figures: [https://journals.plos.org/plosone/s/figures#loc-tools-for-figure-preparation](https://journals.plos.org/plosone/s/figures%22%20%5Cl%20%22loc-tools-for-figure-preparation%22%20%5Ct%20%22_blank).

NAAS will assess whether your figures meet our technical requirements by comparing each figure against our figure specifications.

*In compliance with data protection regulations, you may request that we remove your personal registration details at any time.*  [*(Remove my information/details)*](https://track.editorialmanager.com/CL0/https:%252F%252Fwww.editorialmanager.com%252Fpone%252Flogin.asp%253Fa=r/1/010f019d3cbf1d3a-7c11f965-861f-416b-96a2-e1826a12c1b3-000000/Boa8eI8ih9VDUMfV7l9O5uyqTyAF_sNZUr4-jehuwMs=256%22%20%5Ct%20%22_blank)*. Please contact the publication office if you have any questions.*
